# Human Cardiac Microtissues Display Improved Engraftment and Survival in a Porcine Model of Myocardial Infarction

**DOI:** 10.1007/s12265-025-10596-0

**Published:** 2025-03-13

**Authors:** Evelyne Demkes, Aina Cervera-Barea, Patricia Ebner-Peking, Martin Wolf, Sarah Hochmann, Amy S. Scheren, Mayke Bijsterveld, C. Marlies van Oostveen, Marlijn Jansen, Joyce Visser, Wiebke Triebert, Caroline Halloin, Johannes G. G. Dobbe, Judith de Vos, Melanie Schürz, Joachim Danmayr, Maurice C. G. Aalders, Gerard J. J. Boink, Klaus Neef, Dirk Strunk, Robert Zweigerdt, Saskia C. A. de Jager, Joost P. G. Sluijter

**Affiliations:** 1 Experimental Cardiology Laboratory, Department of Cardiology, University Medical Center Utrecht, Utrecht University, Utrecht, The Netherlands; 2 Regenerative Medicine Center Utrecht, University Medical Center Utrecht, Utrecht University, Utrecht, The Netherlands; 3 Department of Cardiology, Division of Heart and Lungs, University Medical Center Utrecht, Utrecht University, Utrecht, The Netherlands; 4 Cell Therapy Institute, Paracelsus Medical University, Salzburg, Austria; 5 Leibniz Research Laboratories for Biotechnology and Artificial Organs, Hannover Medical School, Hannover, Germany; 6 Cell Therapy Process Development, Novo Nordisk A/S, Maaloev, Denmark; 7 Department of Biomedical Engineering and Physics, Amsterdam University Medical Centers, University of Amsterdam, Amsterdam, The Netherlands; 8 Department of Biosciences and Medical Biology, Paris Lodron University of Salzburg, Salzburg, Austria; 9 Department of Medical Biology, Amsterdam Cardiovascular Sciences, Amsterdam University Medical Centers, University of Amsterdam, Amsterdam, The Netherlands; 10 Department of Clinical and Experimental Cardiology, Amsterdam Cardiovascular Sciences, Amsterdam University Medical Centers, University of Amsterdam, Amsterdam, The Netherlands

**Keywords:** hiPSC-CMs, Cardiac aggregate, Myocardial infarction, Cardiac regeneration, Xenotransplantation, Translational research

## Abstract

Human-induced pluripotent stem cell-derived cardiomyocytes (hiPSC-CMs) constitute a promising therapy for myocardial infarction (MI). The lack of an effective immunosuppressive regimen, combined with single-cell transplantations, results in suboptimal outcomes, such as poor engraftment and compromised therapeutic efficacy. This study aimed to confirm the increased retention of hiPSC-CMs microtissues (CMTs) over single-cell grafts. To ensure the long-term survival of CMTs for potential cardiac applications, CMTs were transplanted in a porcine model of MI using a triple immunosuppression protocol designed to limit immune cell infiltration. Acute evaluation of spherical hiPSC-CMs aggregates and dissociated aggregates followed by the development of a triple immunosuppression protocol were performed in healthy animals. Long-term survival of CMTs was later examined in pigs that underwent a transient coronary occlusion. Two weeks post-MI, the immunosuppression treatment was initiated and on day 28 the animals were transplanted with CMTs and followed for four more weeks. Acutely, CMTs showed superior retention compared to their dissociated counterparts. The immunosuppression regimen led to no organ damage and stable levels of circulating drugs once optimal dose was achieved. Two weeks post-xenotransplantation in healthy pigs, histology revealed that immunosuppressed animals displayed a significant decrease in total cellular infiltrates, particularly in CD3^+^ T cells. Pigs that underwent coronary occlusion, which later were immunosuppressed and treated with CMTs (5 × 10^7^ cells), showed cell engraftment onto the native myocardium four weeks post-transplantation. This study supports the use of a triple immunosuppression cocktail to ensure long-term survival of CMTs for the treatment of MI.

**Graphical Abstract:**

*In vivo* evaluation of CMT transplantation as a regenerative therapy for myocardial infarction. Cardiac microtissues are potential therapies that, when administered in immunosuppressed pigs, have the potential to survive long-term and remuscularize the infarcted myocardium. Figure created with https://BioRender.com.
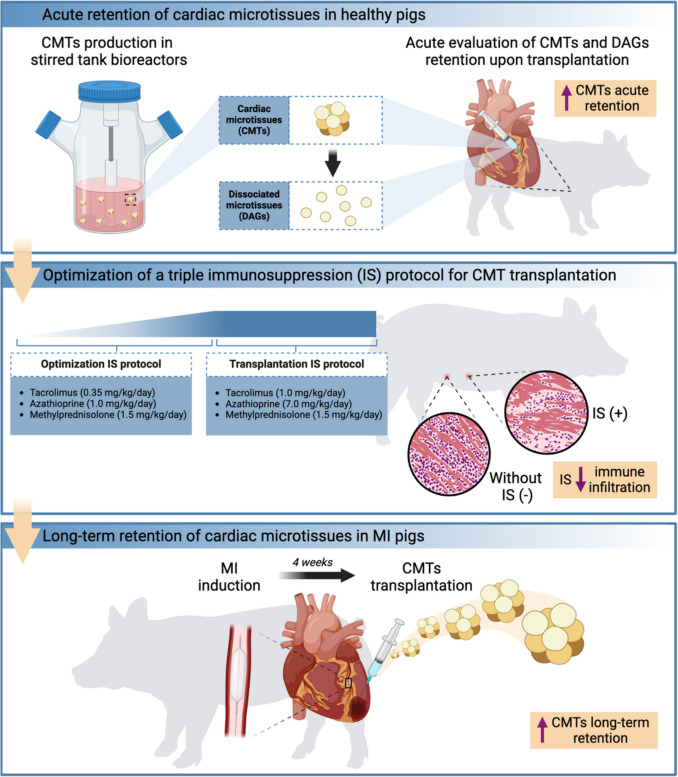

**Supplementary Information:**

The online version contains Supplementary Data available at 10.1007/s12265-025-10596-0.

## Introduction

Ischemic heart disease (IHD) is the leading single cause of death in Europe, taking the lives of almost one million individuals per year [1]. After reperfusion strategies, the currently available treatments only focus on managing post-myocardial infarction (MI) symptoms, attempting to stop the progression into heart failure (HF). As no curative option is yet in place to replace the lost tissue, cell regenerative therapies present a promising approach aiming at heart repair [2]. As a result, post-MI consequences such as cardiomyocyte loss and lack of contractility of the remodeled tissue should be addressed, limiting HF development, and ultimately reducing the need of heart transplants [3].

To date, most promising strategies to replace lost contractile tissue upon exogenous cell injection use bona fide human-induced pluripotent stem cell (hiPSC)-derived cardiomyocytes (CMs) [4]. In this regard, aspects such as cardiomyocyte purity to avoid teratoma formation, large-scale production, cell maturity or capacity to electrically couple to the native myocardium are deemed critical when investigating remuscularization therapies [5]. Nonetheless, one of the biggest challenges that the cardiac cell transplantation field must face is cellular retention upon transplantation. Current retention rates after transmyocardial single-cell delivery have not exceeded 10-15% of transplanted cells in short-term engraftment, regardless of the delivery technique [6–8]. To tackle this, three-dimensional (3D) cell constructs, including microtissues, have been proposed as novel therapies to enhance cell retention, thereby improving cell engraftment and therapeutic efficacy [4, 6, 9–14].

To explore and validate innovative remuscularization interventions with hiPSC-CMs, large animals remain essential to reproducibly model current clinical scenarios. Consequently, the presence of an adequate immunosuppressive regimen is essential to test xenogeneic interventions. General immunosuppression principles have been outlined for clinical application and include a multimodal drug approach against T and B cell-induced rejection. This aims to ensure a balanced immunosuppressive state that should not render patients vulnerable to infections, gradually tapering immunosuppressive doses until maintenance levels are settled [15]. Nonetheless, the current state-of-the-art still debates the most appropriate immunosuppressive drug therapy, leading healthcare providers to establish their own protocols, thereby highlighting once more the absence of consensus in the (pre-)clinical transplantation arena [16–18].

In summary, the present study (graphical abstract) aimed to bridge the gap between xenograft rejection and long-term engraftment of cellular aggregates within the cardiac regenerative domain. Initially, we established a gradual triple immunosuppressive regimen that strongly repressed the immune system. Having a working immunosuppressive cocktail, the assessment of increased cellular retention of large-scale produced hiPSC-CMs aggregates (cardiac microtissues; CMTs) was assessed in a porcine model of myocardial infarction. Here, in the context of an immunosuppression regimen, we report the enhanced and durable retention of CMTs in the infarcted porcine heart.

## Methods

### Production and Culturing of Cardiac Microtissues and Cell-loaded Matrigel® Plugs

Cardiac microtissues (CMTs) were produced by directed differentiation of hiPSC in suspension culture using a stirred-tank bioreactor system, as previously described [19]. In short, after hiPSC expansion in suspension culture, cardiogenic differentiation was induced through the stepwise application of Wnt-pathway modulators. By using glycogen synthase kinase 3 beta (GSK3ß) inhibitor CHIR99201 (CHIR) and the inhibitor of WNT production-2 (IWP-2), we generated highly cardiomyocyte-enriched CMTs within about two weeks of differentiation. See Supplementary Data for details.

For preparation of cell-loaded Matrigel® plugs, CMTs were collected by centrifugation. Matrigel® matrix (850 µL; growth factor reduced (GFR) basement membrane matrix, Corning, 356,231) was mixed with cells and gelated in sterile decapitated syringes containing insoluble suture material (Prolene 4–0 monofil, Ethicon) to increase localization upon removal. Two combinations (in duplicate) were prepared per animal: 1) empty Matrigel® plug and 2) Matrigel® + CMTs (representing ~5 × 10^6^ cells).

### Animal Experiments

#### Animals

All animal experiments were approved by the institutional animal welfare committee from Utrecht University and conducted in accordance with the 'Guide for the care and use of laboratory animals'. Female Topigs Norsvin pigs (Van Beek SPF varkensfokkerij B.V., Lelystad, The Netherlands) were conventionally housed in stables with concrete floor and hay bedding, with light/dark cycles of 12 h and fed standard diet and water ad libitum. Rubber bite sticks were provided as environmental enrichment. See Supplementary Data for details on analgesia, anesthesia, infarct induction, antibiotic prophylaxis and *i.v.* line implantation.

#### Randomization

CMTs were transplanted to test acute retention in healthy animals (CMT, *n* = 4; dissociated aggregates (DAGs), *n* = 3), possible immune rejection in the presence or absence of immunosuppression (immunosuppressed, *n* = 3; non-immunosuppressed, *n* = 2), and long-term survival in infarcted pigs (CMT, *n* = 5; PBS/vehicle, *n* = 3). Researchers were not blinded on treatments and allocated the animals in different groups based on CMT production and availability.

#### Immunosuppression Regimen

During the optimization of the immunosuppression protocol, pigs were treated for 13 weeks with increasing immunosuppression doses and blood was weekly drawn until appropriate circulating levels were achieved. Then, doses were maintained until the end of the study (Fig. 2A). In infarcted animals, immunosuppression was initiated two weeks prior transplantation and continued until the end of the study (Sup. Figure 5). Animals were treated with a triple-drug immunosuppressive regimen consisting of tacrolimus (1.0 mg/kg/day orally; Advagraf, Astellas Pharma), azathioprine (7.0 mg/kg/day orally; Azathioprine Sandoz, Sandoz) and high-dose methylprednisolone (1.5 mg/kg/day *i.v*.; Solu-Medrol, Pfizer). Circulating levels of tacrolimus and azathioprine (6-thioguanine nucleotide (6-TGN) and 6-methylmercaptopurine nucleotide (6-MMP), respectively) were monitored via standard diagnostic testing; methylprednisolone could not be technically measured. Based on established desired circulating levels (tacrolimus 5–20 µg/L; azathioprine (6-TGN) 100–200 pmol/8× 10^8^ red blood cells (RBC), respectively), individual doses were adjusted weekly. Additionally, plasma was collected by whole-blood centrifugation at 1850×g and subsequently stored at -80ºC. Routine clinical chemistry parameters for kidney and liver function were also measured to evaluate potential drug toxicity.

#### CMTs Transplantation in the Heart

At the day of the transplantation, CMTs were collected by centrifugation (300×g, three minutes, room temperature (RT)), washed and resuspended in PBS. After conducting a sternotomy, we stabilized the heart using the Octopus® Evolution (Medtronic, The Netherlands) and administered the CMTs intramyocardially using 25G needles. In healthy hearts, five injections containing each ~ 2 × 10^6^ cells in 200 µL were aimed at the lateral of mid LAD, and special care was taken to avoid injecting close to the coronary venous system. In infarcted hearts, five intramyocardial injections each containing ~1 × 10^7^ in a volume of 200 µL were applied in the border area of the mature scar. We marked the injection sites with suture material (Prolene HEMO-SEAL 5–0, Ethicon) and the thorax was subsequently closed.

### Heart Extraction

Ten minutes after injection (acute) or four weeks post-transplantation (long-term), pigs were euthanized by inducing ventricular fibrillation and the hearts were recovered and processed for further analysis. See detailed Supplementary Data for heart processing, Matrigel® plug processing, and histological analyses.

### Statistical Analysis

All analyses were performed by investigators blinded for group assignments except for immune infiltrate quantifications from HE and CD3^+^stainings. All data are expressed as mean values ± SD. Statistical analysis was done using GraphPad Prism 8.3 with a non-parametric Mann–Whitney test for fluorescent signal data and cell infiltration quantification, Welch’s *t* test for diameter of CMTs in the acute setting, and unpaired *t* test for in vitro proliferation. Statistical analysis for cell viability assay was performed using a one-way ANOVA. A *p*-value ≤ 0.05 was considered statistically significant.

For detailed methods, see the Supplemental Data.

## Results

### Stirred Bioreactors Generated hiPSC-CM CMTs have > 90% CMs Contents

**Fig. 1:**
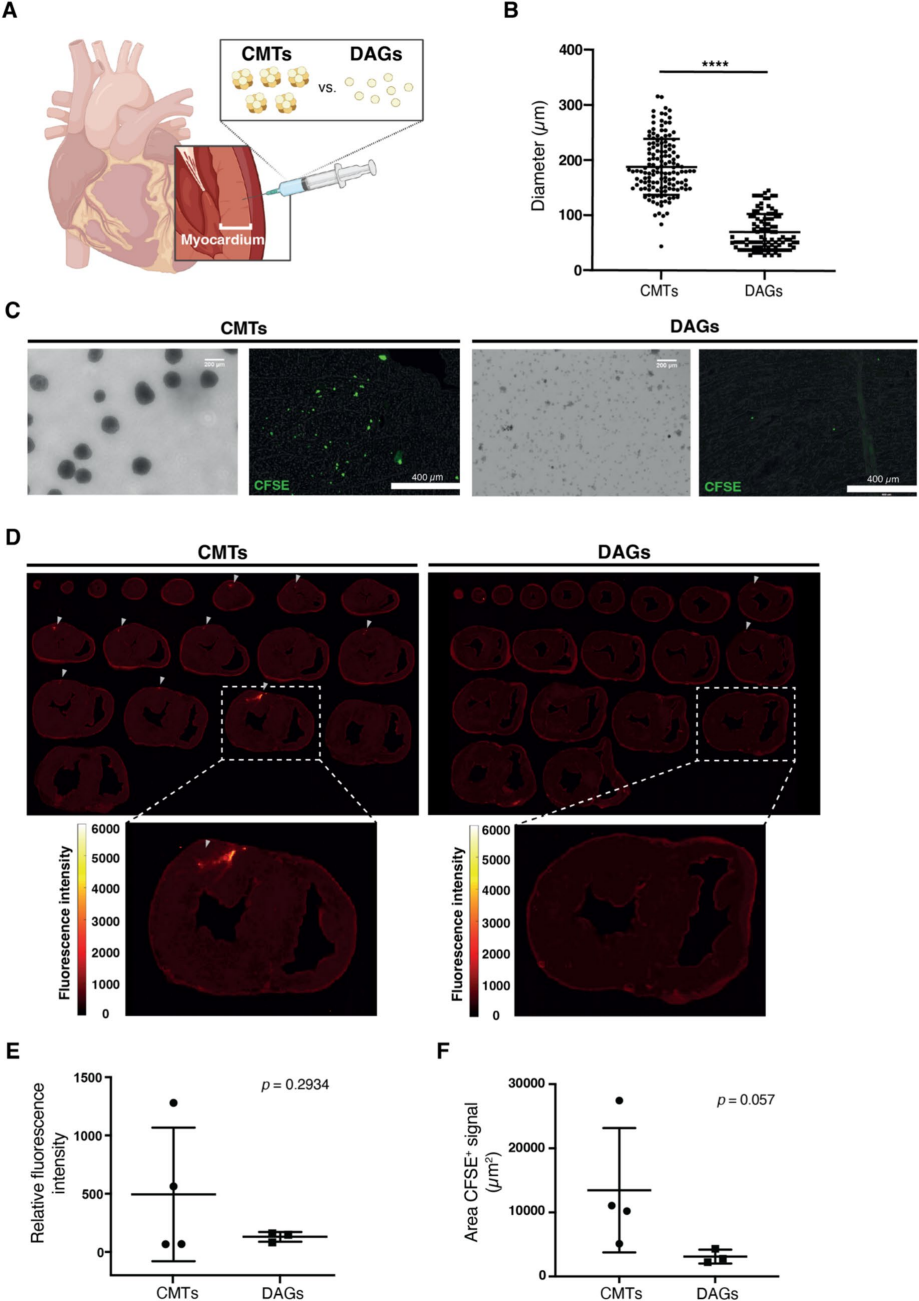
**Comparative assessment of retention between CMTs and DAGs.** **(A) **Schematic representation of the acute cell transplantation study. **(B) **Diameter quantification of CMTs and DAGs. **(C) **Representative bright field images of CMTs and DAGs and histological analysis of pig hearts injection site area of hearts that received CMTs and DAGs. Scale bar bright field = 200 µm; Scale bar histology = 400 µm.** (D) **Whole heart myocardial tissue scans of hearts treated with CMTs or DAGs. Grey arrows point towards the fluorescent CFSE signal in heart slices indicating CFSE signal present in hearts receiving CMTs. **(E and F)** Quantification of relative fluorescence of the whole heart myocardial tissue slices and area (µm^2^) of CFSE positive signal intensity in slices from hearts transplanted with CMTs and DAGs. **** *p *< 0.001, diameter of CMTs *versus* DAGs. Panel (A) was created with BioRender.com. Panel (B): unpaired Welch’s *t* test (*n* = 95-142 biological replicates); data are mean ± SD. Panels (D-E): Mann-Whitney test (*n* = 3-4 biological replicates); data are mean ± SD. CFSE: carboxyfluorescein succinimidyl ester; CMTs, cardiac microtissues; DAGs, dissociated aggregates

Purified hiPSC-derived “cardiac microtissues” (CMTs) were previously transplanted in rodents showing enhanced retention [20], whereas their single cell counterparts displayed substantial early loss [21]. To validate the translational value of transplanting CMTs and their improved retention profile, we performed intramyocardial injections into the porcine heart and evaluated the acute cellular retention (Fig. 1A and Sup. Figure 1A). First, CMTs were generated in suspension culture by directed hiPSC cardiomyogenic differentiation in stirred bioreactors as previously described [19, 22]. This resulted in a population of spherical CMT of 188 ± 50.76 µm diameter (Fig. 1B, C left panel and Sup. Figure 1B) having a CM content of >90% based on flow cytometry analysis (Sup. Figure 1C). To directly compare retention of similar cell sources, we dissociated the CMTs into a suspension of single cells and smaller aggregates (termed dissociated aggregates; DAGs) of approximately 70 ± 33.15 µm diameter (Fig. 1B, C right panel). For post-transplantation detection and visualization of the injected cells in the myocardium, DAGs and CMTs were fluorescently labelled with carboxyfluorescein succinimidyl ester (CFSE). CFSE staining penetrated the entire CMTs, including the center core without essential toxicity (Sup. Figure 1B) since cell death did not exceed 10% both at 2-h (5.6 ± 1.0%) and 24-h (8.5 ± 0.9%) after staining resembling levels in unstained CMTs (2-h: 4.1 ± 0.9% and 24-h: 7.7 ± 0.95) (Sup. Figure 1D).

### Injection of CMTs Results into Higher Acute Retention Compared to DAGs

After assessing the dimensions and viability of DAGs and CMTs, cellular retention of the CMTs was then assessed in an acute experiment in healthy porcine hearts. For this, CFSE-labelled CMTs or DAGs were injected intramyocardially at five different locations of the left ventricle and the tissue was recovered 10 min later (Fig. 1A, Sup. Figure 1 A and Sup. video 1). Whole-heart myocardial tissue analysis revealed CFSE signal in hearts injected with CMTs (Fig. 1D, left panel). In contrast, cross sections from hearts transplanted with the dissociated suspension displayed almost no fluorescence (Fig. 1D, right panel). Interestingly, CFSE signal of the DAGs in the transplanted hearts was still more prominent than previously observed upon injections of single-cell suspensions of bone marrow-derived stromal cells [7, 8].

Relative fluorescence intensity quantification of the cardiac tissue slices showed a non-significant, increase in fluorescence intensity in the CMT transplanted hearts (Fig. 1E). The moderate increase was probably caused by a combined effect of the relative thickness of heart slices (~ 3 mm), a high degree of auto-fluorescence of the tissue, and limited laser penetration of the Typhoon laser-scanner. Using high-magnification fluorescence microscopy, the injections sites transplanted with CMTs clearly exhibited higher CFSE signals (Fig. 1C, left panel). Quantification of these injection sites showed a trend towards more CFSE positive fluorescent area in the CMT group compared to DAGs (CMTs: 13.5 × 10^3^ ± 4.8 × 10^3^ µm^2^ *versus* DAGs: 3.1 × 10^3^ ± 0.6 × 10^3^ µm^2^; *p* = 0.057) (Fig. 1F). Comparable results were observed when assessing the total CFSE fluorescence intensity (CMT group: 6.6 × 10^6^ ± 2.1 × 10^6^ vs. DAG group: 2.1 × 10^6^ ± 0.5 × 10^6^, *p* = 0.114) (Sup. Figure 1E). Herewith, we concluded that transplanting CMTs is favorable over smaller or even single cell injections regarding cellular retention.

### Adequate Systemic Levels of Immunosuppressants Were Achieved Using a Clinically Relevant Triple-Drug Immunosuppressive Regimen

**Fig. 2:**
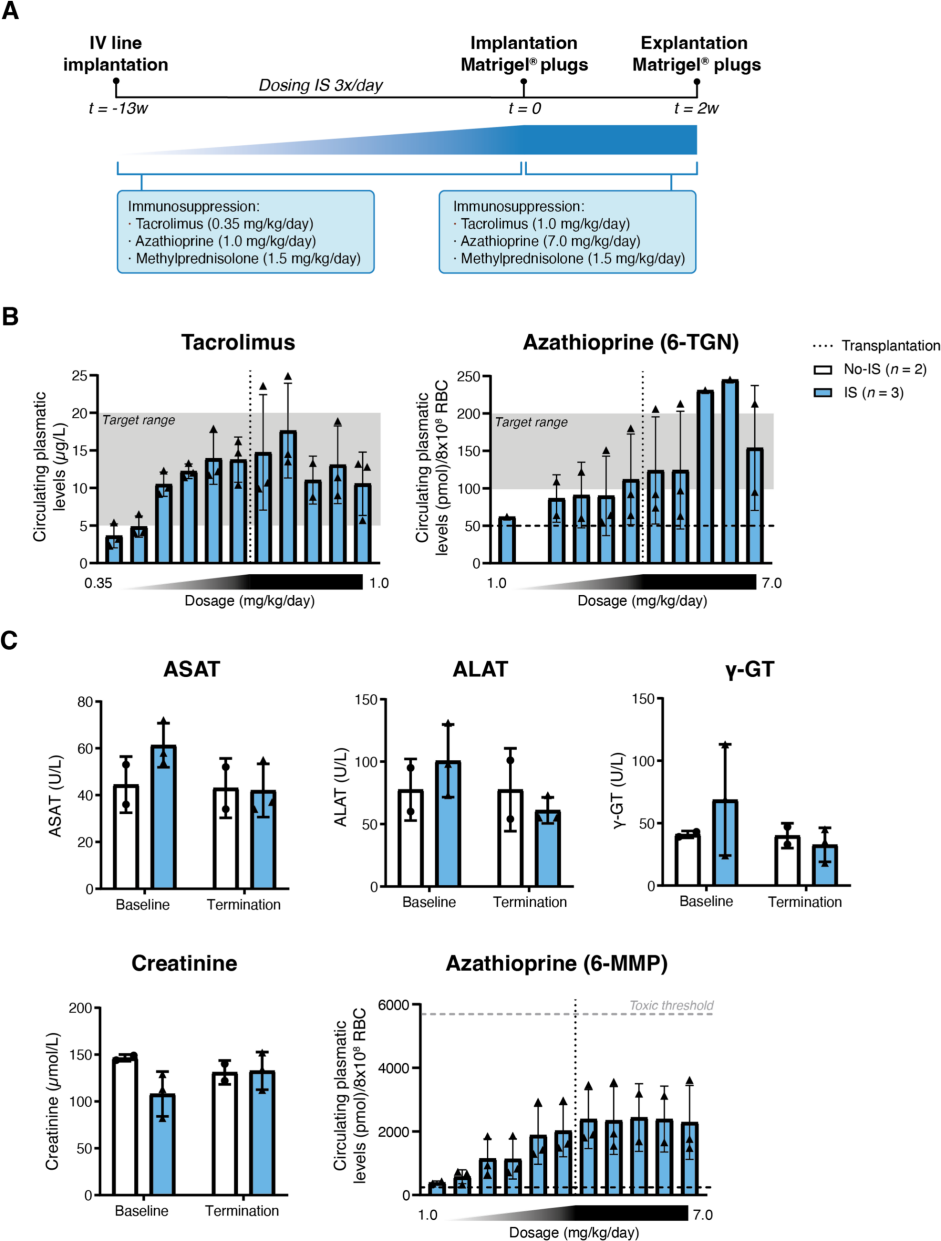
**Overview of immunosuppressive study, drug concentrations, and biochemistry in pigs. (A)** Study design and timeline of the immunosuppressive study. **(B)** Plasma tacrolimus and azathioprine 6-thioguanine nucleotides (6-TGN) concentrations during increased drug dosing and after human donor-cell transplantation. **(C)** Serum concentrations of routine clinical chemistry parameters for kidney and liver function at start of immunosuppressive treatment (baseline) and termination. Azathioprine 6-methylmercaptopurine nucleotides (6-MMPs) levels were also measured during increasing drug dosing and after xenogeneic cell transplantation. Panels (B–C): No-IS (*n *= 2 biological replicates); IS (*n *= 3 biological replicates). Data are individual values ± SD. ALAT, Alanine Aminotransferase; ASAT, Aspartate Aminotransferase; IS, immunosuppression; *i.v.*, intravenous; RBC, red blood cells; t, timepoint; w, week; No-IS, non-immunosuppressed; IS, immunosuppressed; ɣ-GT, Gamma-Glutamyl Transferase

Xenograft rejection is an additional major concern when human-derived cells are tested in clinically relevant large-animal models. To reach stable, systemic immunosuppressive concentrations, equivalent to levels established for human patients, pigs were subjected to a triple-drug immunosuppressive regimen consisting of tacrolimus, azathioprine and methylprednisolone (Fig. 2A). Starting doses of immunosuppressive drugs, equivalent to commonly used concentrations in clinical practice, were not sufficient to reach target concentrations in plasma and required an increasing dose until sufficient levels were reached (Fig. 2B and Sup. Table 2). Blood levels showed that all treated animals achieved circulating tacrolimus levels of 5–20 µg/L after increasing the dose to 1 mg/kg/day (Fig. 2B and Sup. Table 2). As an inactive prodrug, azathioprine requires conversion into the active metabolite 6-thioguanine nucleotides (6-TGNs) to exert its function [23]. One out of three animals reached the aimed human target range of 100–200 pmol/8 × 10^8^ RBC for the active metabolite 6-TGN, whereas the other two animals approached levels at the borderline when administered 7.0 mg/kg/day (Fig. 2B and Sup. Table 2). Circulating methyl-prednisolone levels could technically not be measured, and therefore, dosing remained constant (1.5 mg/kg/day).

High levels of immunosuppressive drugs are also associated with adverse effects, such as the increased risk of hepatoxicity in humans via the formation of 6-methylmercaptopurine nucleotides (6-MMPs) when treated with azathioprine [23]. In consequence, clinical chemistry parameters were monitored to ensure doses remained below toxic reference values (Fig. 2C and Sup. Table 3). Renal and hepatic enzymes were examined in the immunosuppressed pigs, revealing values within the physiological range at baseline and termination, similar to control animals, indicating that the immunosuppressive cocktail did not negatively affect kidney and liver function (Fig. 2C and Sup. Table 3).

**Fig. 3:**
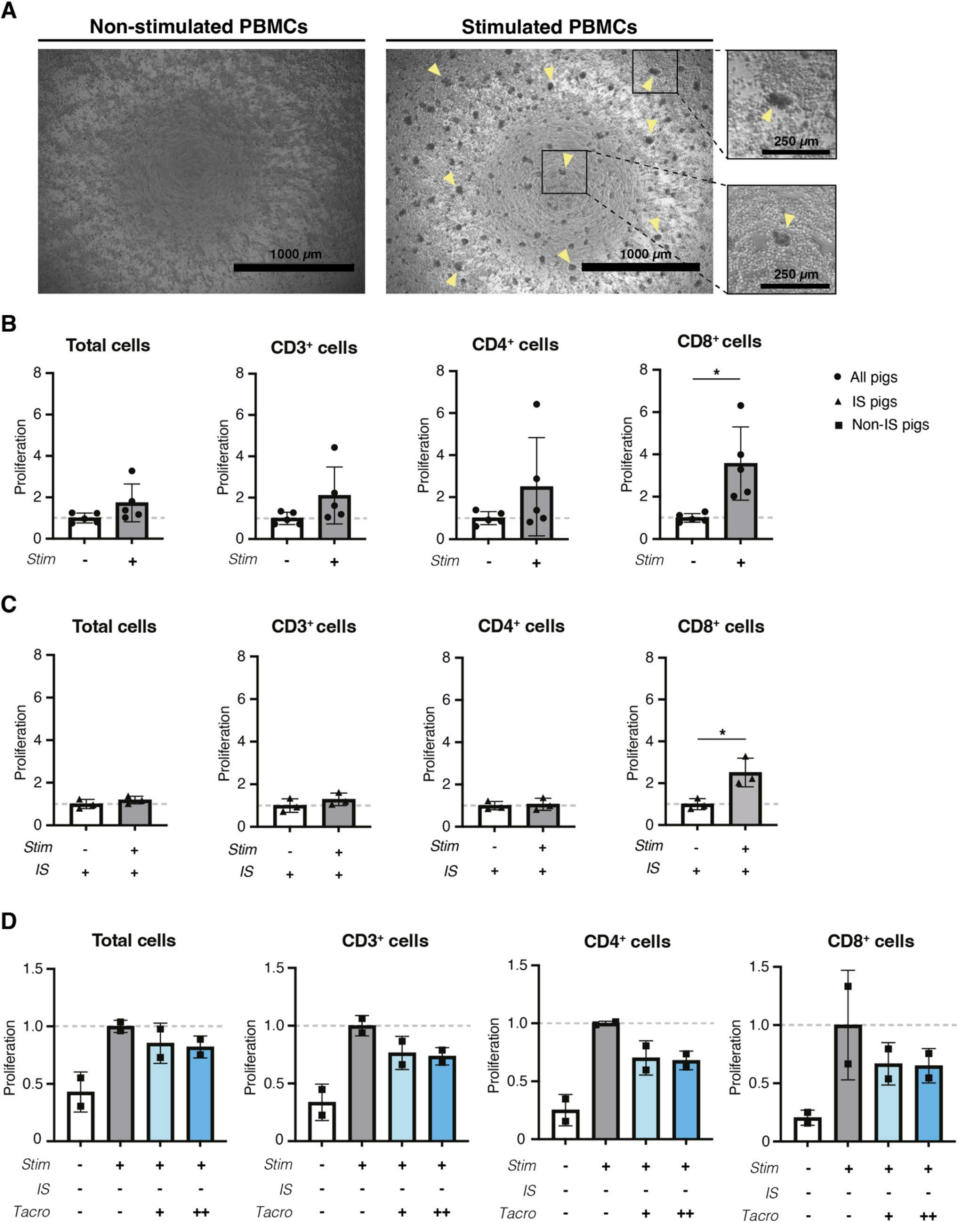
**Porcine PBMCs proliferation profiles after** **in vitro** **stimulation. (A) **Light microscopy representation of non-stimulated (left) or ConA- and IL-2-stimulated porcine clustered-PBMCs (right; yellow arrows) after three days. Scale bar micrograph = 1000 µm; scale bar zoom-in = 250 µm. **(B)** Ratio of total cell proliferation and T cell subsets of non-stimulated and stimulated porcine PBMCs after three days. **(C)**Ratio of total cell proliferation and T cell subsets of non-stimulated or stimulated porcine PBMCs from immunosuppressed (IS) animals. **(D)** Ratio of total cell proliferation and T cell subsets of stimulated porcine PBMCs from non-IS animals incubated with *in vivo*-relevant (Tacro +, 20 ng/L) or high concentrations of tacrolimus (Tacro ++, 100ng/L). Panels (A–C): unpaired *t* test in IS (*n* = 3 biological replicates) and non-IS (*n* = 2 biological replicates). In (B), * *p* < 0.05, CD8^+^ proliferation in stimulated *versus* non-stimulated PBMCs. In (C), * *p* < 0.05, CD8^+^ proliferation of stimulated *versus* non-stimulated PBMCs from IS pigs. Data are mean ± SD. IS (-), non-immunosuppressed; IS (+), immunosuppressed; stim (-), non-stimulated; stim (+), stimulated

An evaluation of an adequate immunosuppression was further assessed in vitro in isolated PBMCs from transplanted animals (with and without immunosuppression). PBMCs were stimulated with ConA and IL-2 for three days. Upon stimulation, PBMCs formed proliferative leukocyte clusters resulting in higher numbers of total cells and specifically for CD3^+^, CD4^+^ and CD8^+^ subsets after normalizing to unstimulated controls, significantly increasing in CD8^+^ (*p* = 0.0109), as analyzed with flow cytometry (Fig. 3A-B and Sup. Fig. S2). PBMCs from immunosuppressed pigs were much less susceptible to stimulation (non-immunosuppressed data not shown), but a significant increase in CD8^+^ cells was also observed after normalization to unstimulated controls (*p* = 0.0232) (Fig. 3C). PBMC activation and proliferation of non-immunosuppressed control animals was typically obtained by normalizing to stimulated animals, and subsequently repressed by adding tacrolimus in a dose equivalent to the in vivo concentration (20 µg/L) (Fig. 3D), while a higher dose (100 µg/L) did not further reduce proliferation (Fig. 3D).

### Limited Immune Cell Infiltrate and Enhanced Human Donor-Cell Survival in Triple-Drug Immunosuppressed Animals

Once relevant triple-drug immunosuppression levels were reached, we implanted high-purity CMTs (Sup. Figure 3) using a GFR Matrigel® carrier in the abdominal area of both immunosuppressed and non-immunosuppressed control pigs (Sup. Figure 4 A and Sup. video 2).

**Fig. 4:**
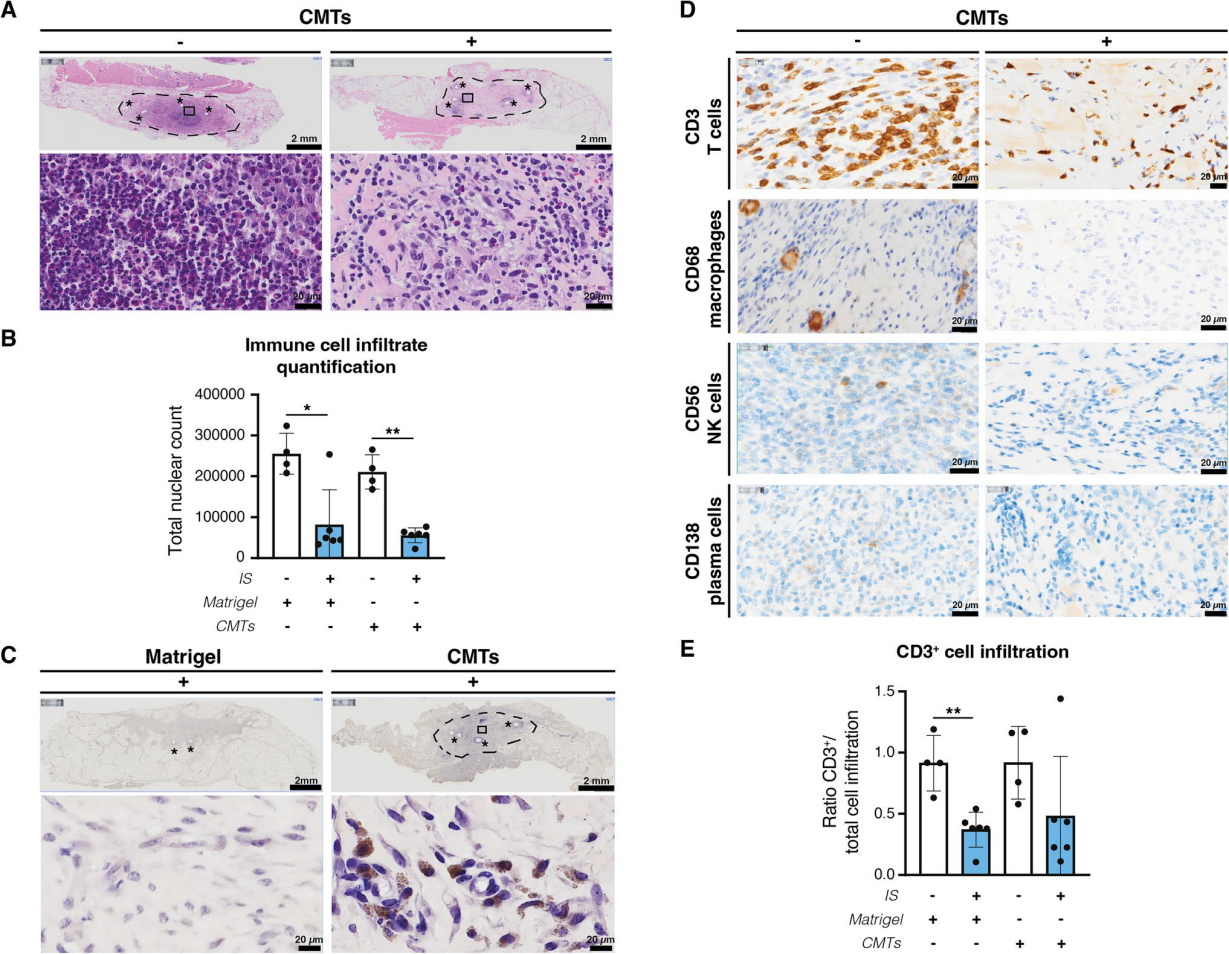
**In vivo detection of CMTs and host cell infiltrate upon transplantation. (A)** Hematoxylin/Eosin staining (HE) of sectioned Matrigel^®^ plugs followed by full scan microscopy visualized the immune cell infiltrate in the CMT-transplanted areas (dashed circle). Plugs isolated from immunosuppressed (IS) pigs revealed limited infiltrate compared to plugs from non-immunosuppressed (No-IS) animals. Scale bar top section = 2 mm; Scale bar magnification = 20 µm. **(B)** Assessment of total cell infiltration via nuclear count quantification in HE stainings after plug implantation. **(C)** Anti-human HLA class I staining (brown) of plugs from IS pigs demonstrated acceptance of the CMTs two weeks after transplantation. Scale bar top section = 2 mm; Scale bar magnification = 20 µm.**(D)** Specific host cell infiltrates in CMT-loaded Matrigel^®^ plugs isolated from No-IS pigs and IS ones. Scale bar = 20 µm. **(E)** Quantification of CD3^+^ cell infiltrates in implanted plugs. Panels (B) and (E): Mann-Whitney test in No-IS *versus* IS (*n* = 2 technical replicates per animal). In (B), * *p* < 0.05, cellular infiltration in Matrigel^®^ only from No-IS *versus* IS pigs and ** *p* < 0.01, cellular infiltration in CMTs from No-IS *versus* IS pigs. In (E), ** *p* < 0.01, CD3^+^ infiltrate in Matrigel^®^ plugs from No-IS *versus* IS pigs. Data are mean ± SD. Asterisk, insoluble suture material hole; black square, zoomed area; dashed line, location of Matrigel^®^ plug; IS (+), immunosuppressed; No-IS (-), non-immunosuppressed

Two weeks after transplantation, the Matrigel® plugs showed macroscopic ulcerous lesions in non-immunosuppressed animals, as confirmed by histology (Sup. Figure 4B). Meanwhile, only moderate immune cell infiltrates were observed in the immunosuppressed pigs (Fig. 4A, right panel) and this infiltration was mostly detected surrounding the suture material (Fig. 4A, asterisks) used to demarcate the transplantation site. The effects of our immunosuppression regimen were further verified by visualizing total cellular infiltration in HE stainings (Sup. Figure 4C-G), which was significantly reduced in immunosuppressed pigs, both in Matrigel®-only (*p* = 0.0381) and CMT-transplanted (*p* = 0.0095) pigs, compared to non-immunosuppressed animals (Fig. 4B).

Two weeks post-transplantation, we deemed the immunosuppressive treatment successful after detecting the human xenografts in examined animals, which displayed HLA class I-positive cells in the CMT group (Fig. 4C). Extensive histological characterization revealed an infiltration of CD3^+^ T cells in CMT-transplanted animals (Sup. Figure 4H-L), with almost an absence of macrophages (CD68^+^), natural killer (NK, CD56^+^) and plasma (CD138^+^) cells (Fig. 4D). Further quantification of the CD3^+^ cellular infiltrate in both CMT and empty Matrigel® plugs, showed a reduction in CMT transplants, albeit only significant in Matrigel® plugs (*p* = 0.0095) (Fig. 4E). Jointly, this indicates that our immunosuppression regimen appears to be suitable for successful xenogeneic cell transplantation in pigs.

### CMTs Show Long-Term Retention and Survival in the Infarcted Porcine Heart

**Fig. 5:**
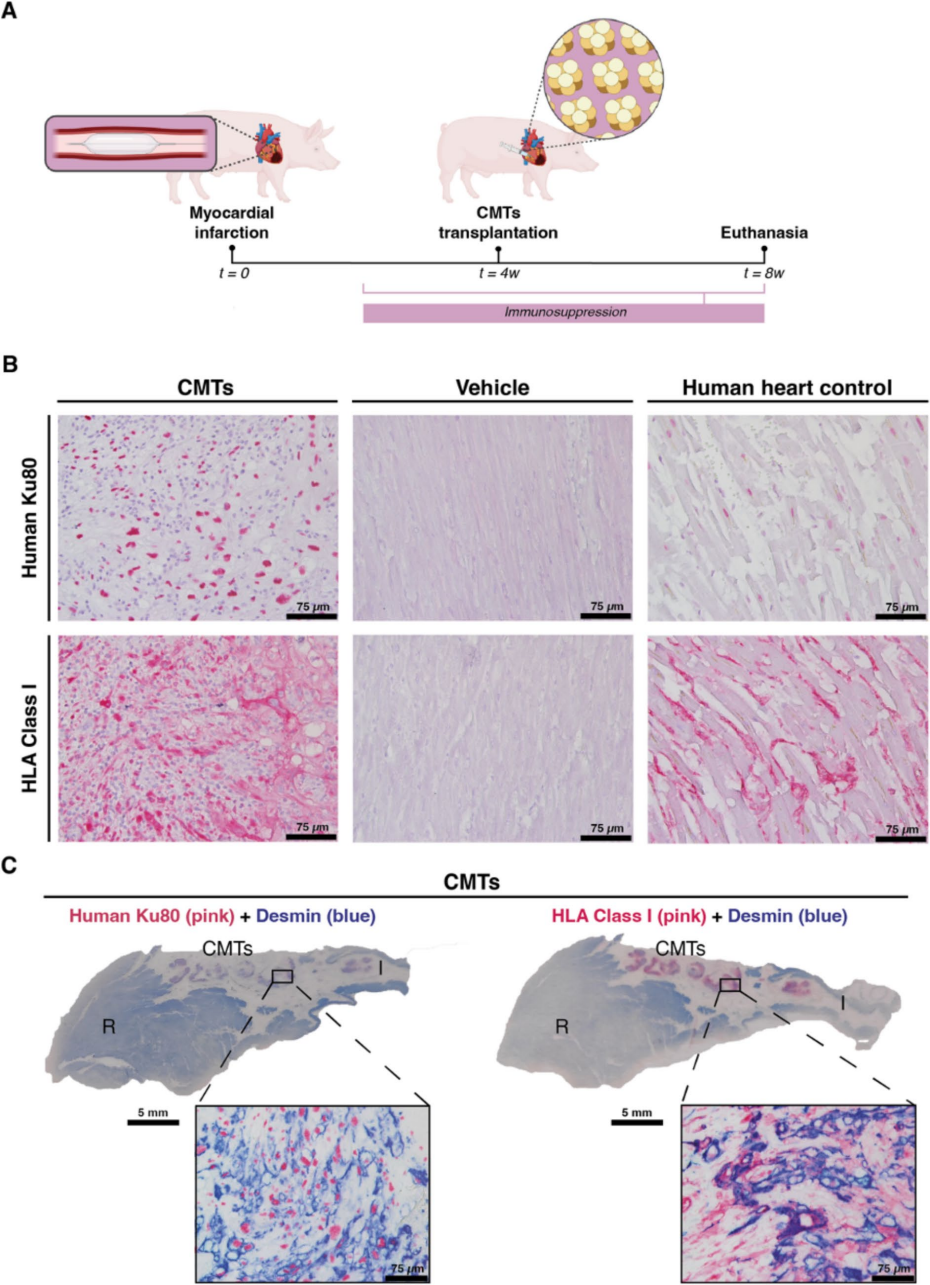
**CMTs detection in the infarcted myocardium four weeks after xenotransplantation. (A) **Schematic representation of the long-term CMTs retention study in infarcted and immunosuppressed pigs. **(B) **Presence and absence of human cells in the infarcted porcine heart four weeks after xenotransplantation of cardiac CMTs or vehicle, respectively. Indicated by human-specific markers Ku80 nuclear antigen (pink) and HLA Class I (pink). Scale bar micrograph = 75 µm. **(C) **Cross-section of CMTs-recipient heart co-stained with human nuclear marker Ku80 (pink) or HLA Class I (pink) and cardiac marker desmin (blue) visualizes large areas of grafted tissue within the infarcted area. Scale bar heart section = 5 mm; scale bar micrograph = 75 µm. Micrographs are representative images from CMT-transplanted (*n* = 1 biological replicate), vehicle (*n* = 1 biological replicate) and human control (*n* = 1 biological replicate) hearts. Panel (A) was created with BioRender.com. CMTs, cardiac microtissues; I, infarcted myocardium; R, remote myocardium

The central hypothesis of this study was to address if CMTs show an improved long-term retention. To assess the long-term retention and survival of the CMTs in the context of cardiac disease, we induced a myocardial ischemia/reperfusion injury in eight pigs by placing an occluding coronary angioplasty balloon mid-LAD for 90 min, followed by reperfusion (Fig. 5A). Optimized triple immunosuppressive treatment was initiated two weeks post-infarction and animals were transplanted with hiPSC-CM constructs two weeks thereafter (Fig. 5A and Sup. Figure 5A). Prior to CMTs transplantation, all treated pigs reached the target range for tacrolimus (> 5 µg/L), although none showed azathioprine’s active metabolite 6-TGN levels within the therapeutic window (> 100 pmol 6-TGN/8 × 10^8^ RBC) (Sup. Figure 5B and Sup. Table 4). Immunosuppression levels steadily increased during follow up, reaching clinical human target ranges in two out of eight animals at the time of termination (Sup. Table 4). All serum concentrations of creatinine, gamma-GT (γ-GT), aspartate aminotransferase (ASAT) and alanine aminotransferase (ALAT) measured prior to termination were comparable to baseline values (Sup. Figure 5C and Sup. Table 5), indicating immunosuppression did not affect kidney and liver function.

At the day of the transplantation, pigs were randomized, and we delivered intramyocardially a dose of CMT equivalent to about ~ 5 × 10^7^ cell in 1 mL PBS/vehicle via intramyocardial injection in the border zone region. Specifically, three batches of CMTs were produced consisting of 144.6 ± 81.5 µm diameter (Sup. Figure 6A and Sup. Figure 6B) with a CM purity of 80–95%, as determined by low cytometry for cardiac troponin T (cTnT), myosin 4 (MF20) and sarcomeric α-actinin (SA) (Sup. Figure 6C).

Four weeks post-transplantation, all animals showed complete transmural infarcts as indicated by picrosirius red staining (representative image, Sup. Figure 6D). Interestingly, in three out of four histologically examined animals, human HLA class I-positive cells were located within the infarcted area (Fig. 5B, C and Sup. Figure 7), indicating CMT retention and cell survival up to four weeks in the recipient hearts (Fig. 5B and C). In fact, the contoured islands found in the largest graft were also positively stained by the human Ku80 nuclear antigen. Co-staining of Ku80 and HLA Class I with desmin confirmed that the identified cell clusters belonged to human muscle cells (Fig. 5C) that remained CMs after being transplanted (Sup. Figure 8). More importantly, large grafts were readily identified in one animal with islands measuring more than half a centimeter in their greatest dimension and, in total, occupying about 10% of the infarcted area. This finding suggested that CMTs reside within the scar tissue and cluster together. When examining other hearts, we could only trace back HLA class I-positive cells in two more pigs (out of four) and the dimensions of these grafts were considerably smaller (pig #1: ~ 5 mm *versus* pig #3: ~ 800 µm *versus* pig #6: ~ 300 µm) (Sup. Figure 7).

**Fig. 6:**
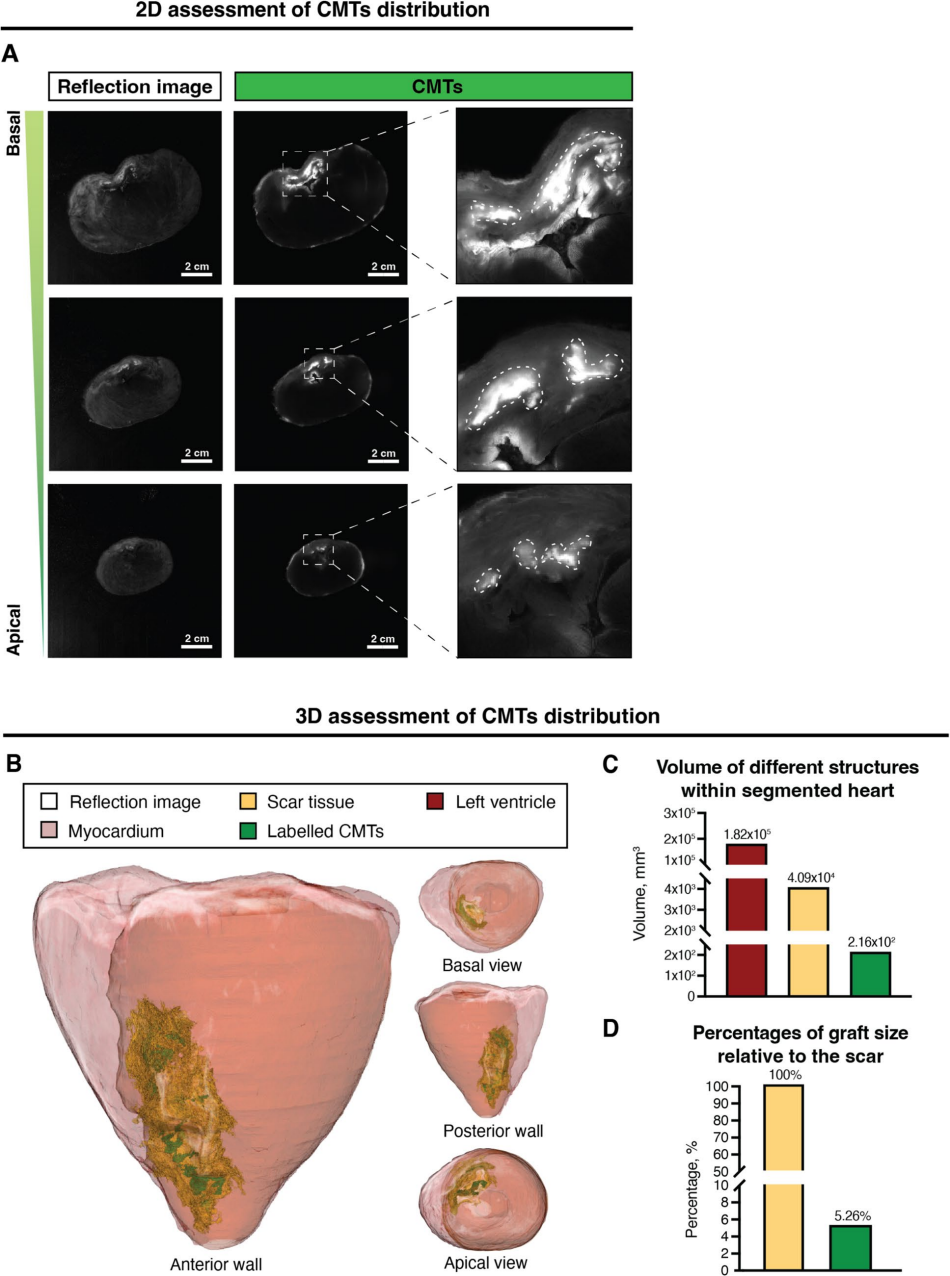
**3D visualization of fluorescently labeled CMTs in an infarcted pig. (A) **Representative macroscopic images of transplanted areas in a heart, from base to apex (top to bottom). Reflection images (left panel) display autofluorescence from the remodeled tissue, together with the fluorescently labelled cells. Upon excitation, xenotransplanted CMTs (right panel) stand out from the remodeled and remote myocardium, indicating localization of xenografts (dashed line). Scale bar = 2 cm.** (B) **Stack of 3350 cross-sectional images of a transplanted heart enabling 3D reconstruction of the transplanted heart.** (C)** Total volume quantification of left ventricle, infarcted tissue, and labelled-CMTs and **(D)** relative contribution of graft in the scar area of the 3D reconstructed myocardial mesh. Representative images and data are single values from a CMT-transplanted heart (*n* = 1 biological replicate). CMTs, cardiac microtissues

To confirm the presence of CMTs and better understand cell biodistribution, one of the porcine hearts was used to generate a 3D replica of the grafts (Fig. 6 and Sup. video 3). The islands of cells detected were on the order of ~ 1 cm (Fig. 6A, right panel), similar in size to what human-specific markers revealed via histology in one animal (Fig. 5C). After discriminating the fluorescent signal, cardiac grafts accounted for 5.26% of the total scar area (Fig. 6D), representing approximately 200 µL of the segmented structures in the heart (Fig. 6D), and potentially reflecting the reduced number of total cells initially transplanted (5 × 10^7^ cells/animal). Looking closely at the distribution of the CMTs in this 3D reconstruction (Fig. 6B), CMTs appeared to be engrafted at the injection sites within the scar tissue, and in clusters bigger than their original injection dimensions, similarly to what histology suggested. These results demonstrate that CMTs were retained long-term, showing the tendency to cluster together, and were readily visible within the infarct scar four weeks post-transplantation.

Finally, despite the optimized immunosuppression regimen applied, we wanted to confirm the potential immunogenic reaction of the grafts four weeks post-transplantation. HE staining confirmed a general cell infiltration in the myocardium, which was prominently found surrounding the grafts, in contrast to animals treated with vehicle (Sup. Figure 9A). As a matter of fact, this cellular infiltration positively stained for a pan-leukocyte marker CD45 and CD107a which is expressed on activated macrophages, NK cells and CD8^+^ cytotoxic T cells (Sup. Figure 9B). Coincidentally, three of the CMT recipients that presented long-term engraftment and a positive immune infiltration surrounding the CMTs did not reach azathioprine target levels at termination (Sup. Table 4). Nonetheless, human reference values were used for the immunosuppression treatment and thus it should not necessarily question the efficacy of the immunosuppression regimen.

To evaluate the reorganization, possible integration, and maturation stage of the CMTs within the native porcine myocardium four weeks after transplantation, intercalated disk protein N-cadherin was used. A robust expression of N-cadherin was present in human hearts, being much lower and disorganized in the in vivo long-term transplanted porcine heart. Moreover, the lack of circumferential disposition of the intercellular junctions suggested cardiomyocyte immaturity (Sup. Figure 9C) [24].

## Discussion

The aims of our study were 1) to optimize and validate a stepwise immunosuppression protocol that would allow the long-term evaluation of xenotransplanted human grafts, and 2) combine this approach with the enhanced short and long-term retention of CMTs in healthy and infarcted pigs, respectively. The applied CMTs were generated with the use of stirred bioreactors [19, 22] enabling a scalable production relevant from a clinical standpoint, which can be adapted to GMP-compliance [25].

Improved longevity of non-autologous grafts is largely dependent on an adequate immunosuppression regimen implemented, which is even more so important for xenogeneic grafts [26]. In the clinical setting for solid organ transplantation (SOT), maintenance regimens generally consist of a combination therapy with calcineurin inhibitors (CNI), antiproliferative agents and glucocorticosteroids (GCS) [27]. Additionally, acute and chronic inflammatory responses play a pivotal role in the healing process after ischemia and during cardiac remodeling [28]. Hence, it is utterly important to find a balance between avoiding graft rejection and not interfering with the healthy remodeling process. We therefore applied a stepwise increased dosing and weekly testing to reach stable immunosuppressive plasma concentrations equivalent to levels established for human patients. The absence of kidney and liver failure suggests that the current immunosuppressive approach is safe. Nonetheless, our starting doses of immunosuppressive drugs commonly used in clinical practice were not sufficient to reach human reference target concentrations and required increasing doses until threshold therapeutic levels were achieved in some animals.

Maintenance anti-rejection therapy remains center-specific but the combination therapy of CNI, antiproliferative drugs and GCS aims at minimizing immune-mediated graft rejection by downregulating cytokine production, interfering with effector T-cell synthesis and inhibiting prostaglandin-mediated inflammation [29]. Triple-drug regimens are given at lower dosages compared to induction which aids curbing medication-related side effects, albeit they still come with vexing therapeutic problems. Firstly, there is no evidence of the lowest effective immunosuppressive dose, therefore surveillance of graft integrity together with drug plasma levels and side effects is required. Additionally, dyslipidemia, hypertension, diabetes, malignancy or organ toxicities are among the most common complications [30–32]. Subsequently, doses might need to be re-adjusted, brief course of GCS must be initiated, or even in some instances agents must be replaced or discontinued altogether [33]. Nonetheless, triple-immunosuppressant regimens are the most common maintenance approaches at discharge, endorsed and adopted by various relevant health organisms [27, 34, 35], thus we wanted to follow the recommendation of experts in this study.

Zhu and colleagues already confirmed that a multi-drug regimen improved survival and engraftment of transplanted cells in infarcted hearts over a cyclosporine A (CsA) monotherapy [36]. Nowadays, many other translational researchers are implementing such triple drug therapies in pigs and non-human primates (NHPs). The cocktails vary per study, being in the recent years mostly a combination of CsA, abatacept and methylprednisolone used to warrant xenograft success [13, 14, 37–39]. In this study, we wanted to ensure not only graft survival but also drug tolerance. Unfortunately, we were unable to reach plasma target levels for CsA (data not shown), therefore we switched to tacrolimus. The dose choice (1 mg/kg/day), together with azathioprine’s, was motivated by our dose-finding study which coincided with Kawaguchi’s tacrolimus dose, unpublished by the time this study was conducted [11]. Moreover, the preclinical field had not yet adopted abatacept, and combined with the high costs of mycophenolate mofetil, we were inclined to use azathioprine instead, known to be a traditional yet robust immunosuppressant for anti-rejection therapy [29, 40, 41]. All in all, we believe that the immunosuppressive regimen for this work was a sound choice to treat the animals and in line with the current triple-drug regimens used in similar studies.

Once the immunosuppressive treatment was validated, we proceeded with assessing its cell retention efficacy in a highly immunogenic transplantation site, being the abdominal subcutaneous layer as one of the most robust immunologic responses possible [42, 43]. Immune reactions against non-autologous cell transplants are primarily instrumented by an acute cellular rejection driven by T-cell alloantigen recognition [44]. In our study, significant reduction of CD3^+^ infiltrates was only visible in empty Matrigel® plugs from immunosuppressed pigs, in combination with limited number of other infiltrated host immune cell types. The guidelines for the diagnosis of heart rejection categorize these infiltrates as mild acute cellular rejection, which can even resolve with any further treatment posing a relatively no threat to the CMT recipient although close monitoring is always advised [45].

Moving forward, we showed that four weeks after transplantation, and in combination with immunosuppression, large areas of CMTs could still be recognized within the infarcted porcine myocardium in one pig. In two others, the findings were less prominent in size, but the minimal presence of HLA class I-positive infiltrates revealed the retention of small islets of cells in CMT recipients. Immune cell infiltration was present, as demonstrated by the influx of CD45^+^ and CD107a^+^ cells, but whether these infiltrates originate solely from an inflammatory rejection or from a wound-healing response, as recently reported by Vagnozzi, et al. [46], that remains unclear.

In previous studies, therapies intending to deliver single cells culminated in limited acute engraftment [7, 8], resulting in almost absent grafts by the time of termination. Based on findings from Hattori et al. [9], we anticipated an increased retention via cell aggregates, and hereby could confirm that, at a scale in the order of millions of cells, CMTs displayed a superior retention upon intramyocardial injection. In this respect, transplanting larger cell constructs has proven to be advantageous in increasing retention [6, 8, 10, 14, 47, 48], together with immunosuppression implementation.

Not only cell aggregates are crucial to increase retention, but they might also reduce the total cell numbers needed for transplantation. To make up for the myocyte deficit, high doses of human CMs (up to one billion cells) have been injected intramyocardially in NHPs and pigs in an attempt to induce remuscularization of the infarcted area [14, 16, 49, 50]. In such studies, surviving grafts occupied ~ 5-18% of the scar area [16, 49, 51]. In the current work, by injecting only 5 × 10^7^ cells, we demonstrated the possibility of considerably reducing cell numbers when transplanting CMTs to achieve cell graft retentions in a similar range. Previously, aggregations of hiPSC-CMs with gelatin hydrogel proved long-term functional improvements in intramyocardially transplanted micro-pigs, but not after intracoronary delivery of spheroids in NHPs [13]; the increased cell retention in both species was difficult to validate long-term [11]. Presently, these findings are being translated in a first-in-human study (NCT04945018) [52], where the aim is to assess the efficacy of clustered hiPSC-CMs in the context of severe HF, secondary to IHD. In our current proof-of-concept study, remuscularization of the native damaged myocardium was never intended, yet the presence of CMTs four weeks post-transplantation accounted for one fifth of the total volume injected. Although these volumes should not be taken literally, they represent aggregate tissue distribution and are indicative of how many cells would be needed to remuscularize the scarred tissue. Notwithstanding, these findings together with the current work in the (pre-)clinical arena confirm our initial hypothesis proving that CMTs offer a better retention profile than single-cell preparations, although greater cell numbers are indicated for the latter, and cell washout and possible functional decline cannot be entirely ruled out in the long-term.

When assessing the adhesion capacity of CMTs, we observed disruption and loss of N-cadherin expression four weeks post-transplantation, suggesting an absence of integration with the native myocardium. However, it could also be a manifestation of intracellular reorganization to facilitate further integration, as previously observed throughout cardiac trabeculation during heart development and maturation [53]. Going forward, we propose combining cellular aggregates seeded in 3D constructs to prompt maturation and engraftment of the cells.

Additionally, the inability to structurally couple with host tissue together with the immaturity of transplanted cells, may not only result in decreased contractile function [54], but also trigger engraftment arrhythmias (EAs) [16, 49–51]. In fact, the origin of EAs was recently discovered by Marchiano and colleagues, previously hypothesized by others [16, 49], after transplanting quadruple-edited hiPSC-CMs. The MEDUSA cell line presented in their study abolished depolarizing currents (*HCN4*, *CACNA1H* and *SLC8A1*) and overexpressed hyperpolarizing ones (*KCNJ2*). This enabled the demonstration that EAs stem from grafts’ fetal-like ion channel expression causing an impulse disorder [38], rather than re-entrant loop formation as it was formerly believed. These insights should be relevant in the advent of cardiac regenerative therapies when developing less pro-arrhythmic cell lines.

In conclusion, in this study we showed that upon transplantation of CMTs and an adequate immunosuppressive regimen, long-term cell retention and survival can be gained in a clinically relevant large animal model of MI. As we solely focused on long-term retention and survival, our study was neither powered nor designed to investigate beneficial effects on function, contractility, or arrhythmia incidence. Nevertheless, it indicates the importance of the application of 3D CMs in terms of engraftment and maturity, which might be further enhanced using tissue engineering techniques to maximize restorative results and precipitating translational applicability. Furthermore, efficacy studies should be conducted to test whether CMTs transplantation would mediate functional improvements in an ischemic setting and prevent HF progression.

## Study Limitations

In the current study, we demonstrated the acute increased retention of CMTs compared to their dissociated counterparts, and their long-term engraftment in a porcine heart ischemic model. The study was not designed to assess functional outcomes, such as presence of pro-arrhythmia or LVEF improvement, which are important endpoints to acknowledge in the context of HF. Another aspect to consider is the limited follow-up period, which could be impeding the maturity and integrative assessment of the graft in the long run. Collectively, studies that include higher cell numbers, are less invasive, with an adequate immunosuppression, offering longer follow-up periods and aiming to assess functional recovery are warranted to deem these cardiac transplants adequate therapies for ischemic patients.

## Supplementary Information

Below is the link to the electronic Supplementary Data.

ESM 1 (MP4 32.2 MB)

ESM 2 (MP4 13.4 MB)

ESM 3 (AVI 3.75 MB)

ESM 4 (DOCX 17.9 MB)

## Data Availability

All data are incorporated in the manuscript text and the Supplementary Data.

## Abbreviations

CMTs: Cardiac microtissues
DAGs: Dissociated aggregates
EA: Engraftment arrhythmia
HF: Heart failure
hiPSC-CMs: Human-induced pluripotent stem cell-derived cardiomyocytes
IHD: Ischemic heart disease
LAD: Left anterior descending
LV: Left ventricle
LVEF: Left ventricular ejection fraction

## Declarations

### Generative AI and AI-assisted Technologies in the Writing Process

The authors did not use generative AI or AI-assisted technologies in the development of this manuscript.
