# Supplementary material for: Human Cardiac Microtissues Display Improved Engraftment and Survival in a Porcine Model of Myocardial Infarction
Source: J Cardiovasc Transl Res. 2025 Mar 13;18(3):512–28. doi: 10.1007/s12265-025-10596-0 (PMC12208958; doi:10.1007/s12265-025-10596-0)
Supplement: Supplementary file 4 — (DOCX 17.9 MB) [file 12265_2025_10596_MOESM4_ESM.docx]

**Human cardiac microtissues display improved engraftment and survival in a porcine model of myocardial infarction**

Evelyne Demkes, PhD^1,2,3,#^, Aina Cervera-Barea, DVM MSc^1,2,3,#^, Patricia Ebner-Peking, PhD^4^, Martin Wolf, PhD^4^, Sarah Hochmann, PhD^4^, Amy S. Scheren, MSc^1,3^, Mayke Bijsterveld, MSc^1,3^, C. Marlies van Oostveen, MSc^1,3^, Marlijn Jansen, BSc^1,3^, Joyce Visser, BSc^1,3^, Martijn van Nieuwburg-Fennema, BSc^1,3^, Wiebke Triebert, PhD^5^, Caroline Halloin, PhD^6^, Johannes G.G. Dobbe, PhD^7^, Judith de Vos, BSc^7^, Maurice C.G. Aalders, PhD^7^, Gerard J.J. Boink, MD PhD^8,9^, Klaus Neef, PhD^1,2,3^, Dirk Strunk, MD^4^, Robert Zweigerdt, PhD^5^, Saskia C.A. de Jager, PhD^1,3^, Joost P.G. Sluijter, PhD^1,2,3^

^1^ Experimental Cardiology Laboratory, Department of Cardiology, University Medical Center Utrecht, Utrecht, The Netherlands.

^2^ Regenerative Medicine Center Utrecht, Circulatory Health Research Center, University Medical Center Utrecht, University Utrecht, Utrecht, The Netherlands.

^3^ Department of Cardiology, Division of Heart and Lung, University Medical Center Utrecht, The Netherlands.

^4^ Cell Therapy Institute, Paracelsus Medical University, Salzburg, Austria.

^5^ Leibniz Research Laboratories for Biotechnology and Artificial Organs, Hannover Medical School, Hannover, Germany.

^6^ Cell Therapy Process Development, Novo Nordisk A/S, Maaloev, Denmark.

^7^ Department of Biomedical Engineering and Physics, Amsterdam UMC location, Amsterdam, The Netherlands.

^8^ Department of Medical Biology, Amsterdam UMC location, Amsterdam, The Netherlands.

^9^ AMC Heart Center, Department of Clinical and Experimental Cardiology, Amsterdam UMC location, Amsterdam, The Netherlands.

^#^ Equally contributed.

**Supplementary methods**

**Production and culturing of hiPSC-CM microtissues**

Two human-induced pluripotent stem cell (hiPSC) lines were used for cardiac differentiation in this study: 1) hHSC_Iso4_ADCF_SeV-iPS2 line (“Phoenix”; RRID: CVCL QX51) derived from CD34+ cord blood cells [1] and 2) MHHi0001-A-11 (“Amber”; transgenic line derived from Phoenix), the latter carrying a transgene for constitutive expression of nuclear yellow fluorescent protein (YFP).[2] Amber-derived cardiac microtissues (CMTs) were used in the acute retention evaluation and in the heart examined through cryoimaging from the long-term experiment, whereas Phoenix-derived CMTs were used in Matrigel^®^ plugs and long-term retention in infarcted pigs. After directed hiPSC differentiation, CM purity was evaluated with flow cytometry for the CM-specific markers, cardiac troponin T (cTNT), myosin heavy chain (MF20) and sarcomeric α-actinin (SA). The resulting CMTs were cultured in 125 mL Erlenmeyer flasks (max. 20 million cells per flask) containing 20 mL RPMI 1640+L-glutamine medium (Gibco, 11875093) enriched with 2% B-27 supplement (50X) with insulin (serum free, Gibco, 17504044) and 1% penicillin-streptomycin. The medium was replaced every three days and flasks were continuously kept on an orbital shaker (Celltron, ø25 mm, 230V) at 70 revolutions per minute (RPM) in a cell culture incubator (37°C, 5% CO_2_, 20% O_2_) until transplantation. Prior to transplantation, CMTs were collected by centrifugation at 300 × g for three min in 50 mL conical tubes, washed twice in 10 mL PBS (Gibco), resuspended in one mL PBS and transferred into a sterile one mL syringe with a 25G needle attached.

**CFSE labeling, CMT dissociation and cell viability assay**

*CFSE labeling*

To allow acute donor cell/ CMT visualization post-transplantation, CMTs were labelled with 5μM CFSE (Sigma, 21888) for 20 minutes on an orbital shaker (70 RPM) in a cell culture incubator (37°C, 5% CO_2_, 20% O_2_) in the dark. Later, cells were centrifuged three minutes at 300 × g, washed thrice with PBS and diluted in 10% FBS. Syringes attached to 25G needles were then loaded for injection.

*Dissociation of microtissues*

To generate dissociated aggregates (DAGs) controls, CMTs were incubated with Collagenase A (1 mg/mL in PBS, Roche, 10103586001) in a 37°C water bath for 20 minutes with regular manual mixing. The dissociation was stopped by centrifugation-based cell collection at 300 × g for three min in 50 mL conical tubes, washed twice in 10 mL PBS (Gibco), and stained with CFSE for 10 minutes in the incubator (same conditions as described previously). DAGs were suspended in 10% FBS mL PBS and loaded to a syringe for injection, equivalent to the CMTs.

*Cell viability assay*

Cell viability was assessed by an LDH cytotoxicity assay (Takara, LDH cytotoxicity detection kit, MK401) according to manufacturer’s instructions and analysis was performed using a Multiskan FC microplate Photometer (ThermoFisher Scientific). Percentage of cell death was calculated by data interpolation of Log10 transformed absorption values from known cell death percentages (Sup. Fig. 1E).

**PBMC isolation, stimulation assay and FACS**

*PBMC isolation*

Venous whole blood of immunosuppressed pigs was collected weekly in K2 EDTA-coated vacutainers (BD vacutainer) to later isolate peripheral blood mono-nucleated cells (PBMCs). On the same day of collection and using a gradient density medium (Ficoll-Paque™ PLUS, GE Healthcare Bio-Sciences), we isolated PBCMs by positive selection according to manufacturer’s protocol.

*PBCM culture and stimulation assay*

Isolated PBMCs were cultured (3x10^5^ cells/well seeded in quintuplets in round bottom 96-wells plate) in RPMI medium enriched with 10% heat inactivated fetal bovine serum (FBS) and 1% P/S. Cells were subsequently stimulated in several conditions: Concanavalin A (ConA; 5 μg/mL, Sigma-Aldrich) + Interleukin-2 (IL-2; 100 ng/mL, R&D Systems), ConA (5 µg/mL) + IL-2 (100 ng/mL) + Tacrolimus (Tacro; 20 µg/L, *in vivo* concentration), ConA (5 µg/mL) + IL-2 (100 ng/mL) + Tacro (100 µg/L, abundance) or not stimulated for three days. Later, cells were collected and stained for 30 minutes at 4°C with antibodies (Sup. Table 1) and 7-AAD viability staining solution (420403, BioLegend) for 15 minutes.

*FACS*

After stimulation, cell viability and proliferation were measured by flow cytometry (Gallios, Beckmann Coulter) and analyzed using Kaluza Analysis Software (Beckman Coulter, version 2.1). Gating strategy can be found in Sup. Fig. 2.

**Animal experiments**

*Anesthesia and analgesia*

Pigs were pre-treated orally with amiodarone for 10 days (1200 mg loading dose, 800 mg/day maintenance), clopidogrel for 7 days (600 mg loading dose, 75 mg/day maintenance) and acetylsalicylic acid for 7 days (320 mg loading dose, 80 mg/day maintenance) before the infarct induction and continued until the end of the study. One day before surgery, animals received a buprenorphine patch (5 µg/hour) to ensure adequate analgesia. On the day of the surgery, animals were premedicated with an intramuscular injection of ketamine (15 mg/kg), midazolam (0.4 mg/kg) and atropine (0.05 mg/kg), followed by the intravenous (*i.v*.) administration of thiopental (4 mg/kg) for induction. Animals were intubated and mechanically ventilated with a 1:2 oxygen-air ratio. Maintained anesthesia was achieved with *i.v.* infusion of cis-atracurium (0.7 mg/kg/hour), midazolam (1 mg/kg/hour) and sufentanil (2,5 µg/kg/hour).

*Infarct procedure and intravenous line insertion*

Pigs were subjected to a myocardial infarct by occluding the left anterior descending (LAD) coronary artery with an angioplasty balloon for 90 minutes.[3], [4] After arterial and venous access was obtained, a catheter (8FR JL4 guiding, Mach 1, Boston Scientific) was placed in the left coronary tree and a coronary angiogram was acquired. Subsequently, LAD diameter was measured, an adequately sized balloon was placed mid-LAD and inflated to nominal pressure. At start and 45 minutes after occlusion time, balloon inflation and LAD occlusion was verified with a coronary angiogram. In case of ventricular fibrillation (VF), animals were defibrillated while receiving 150 mg amiodarone bolus *i.v*.. Venous access was gained from the jugular vein canalled to the back of the animals to implant an *i.v.* line for continuous drug delivery and blood sampling. The venous line was protected with a mesh bandage around the thorax to prevent it from being accidentally pulled off and flushed daily with 0.9% NaCl solution followed by heparin-solution (0.1% heparin in 0.9% NaCl solution) to prevent clog formation.

*Antibiotic prophylaxis and mitigation of opportunistic infections*

All pigs received prophylactic *i.v.* broad-spectrum antibiotics (amoxicillin + clavulanic acid) on the surgery day (500mg/kg) and oral tablets two-times/day the day after surgery (12.5 mg/kg/dose). Active monitoring was conducted the first 72 hours post-surgery to detect any signs of inappetence, pain, lethargy, or dyspnea. Additionally, fever onset was monitored by daily rectal temperature measurements until terminal time-points in the dose-finding immunosuppression animals and three-day post-surgery in infarcted pigs. During feeding and cleaning, caretakers would also check the pigs and report to the researchers and designated veterinarian any behavior that deviated from normal. Opportunistic systemic infections were not detected throughout the studies, except for a couple of animals that presented puss where the *i.v.* line was implanted. A bolus of neomycin + penicillin was applied onto the *i.v.* line incision and monitored until it stopped suppurating.

**Heart extraction, processing, and histological analyses**

*Heart excision, processing, and visualization of grafts for acute retention*

Upon completion of the study, animals were sacrificed by administration of potassium chloride. Hearts were then embedded in agarose (agar) in an end-diastolic geometry and left at 4°C for the agar to set overnight.[5] Next, the heart was sliced in ~3 mm heart slices using a Berkel™ Meat Slicer and each individual heart slice was scanned using a Typhoon 9400 scanner (GE Healthcare, Amersham, variable imager, green (532nm) laser and blue (488nm) laser for background control) from both apical and basal side. Analysis was performed using Cellprofiler Software (3.1.8.). Scanning of the heart with a Typhoon scanner illustrated auto-fluorescence of epicardial tissue and heart valves. However, the fluorescence of the CFSE-label could clearly be distinguished from autofluorescence of the heart.

Tissue samples from the injection area were collected and cut into 5 µm slices at 4 different depths to score for the presence of CFSE-labeled cells. Representative slides (*n* = 3) were chosen for each pig and scanned using a Nanozoomer 2.0 (Hamamatsu, Japan). Each slide was analyzed for total area of CFSE+ signal and total signal intensity using QuPath software (v0.1.2).

*Processing of Matrigel^®^ plugs*

Matrigel^®^ plugs were fixed in formalin solution (10%, neutral buffered, Sigma-Aldrich; HT5011), paraffin-embedded and cut into 2 µm slices.

*Heart excision and processing for long-term survival of the grafts*

Upon completion of the study, animals were sacrificed by administration of potassium chloride. Whole heart was extracted, atria and valves were removed, and the heart was fixed in 4%-formaldehyde for 7 days. Afterwards, hearts were transversely sliced in ~10-15 mm slices using a Berkel™ Meat Slicer and fixated in fresh 4%-formaldehyde for seven more days. Next, tissue was processed, embedded in paraffin, and cut into five µm thick slices.

*Histological analyses*

Matrigel^®^ plugs. Hematoxylin/Eosin (HE) staining was performed in a linear slide stainer (Leica ST4040) using Mayer’s Hemalum (1.09249.2500, Merck) and Eosin Y (1.15935.0100, Merck). For immunohistochemistry, sections were deparaffinized for 2x 15 min in Xylene (Sigma-Aldrich) and endogenous peroxidase activity was blocked in Methanol/H_2_O_2_ (Merck) for 30 min. The sections were rehydrated, and non-specific antibody binding was blocked by incubating sections with 10% FCS in Dako buffer (Agilent) for 20 min. Primary antibodies (listed in Sup. Table 1) were applied in 10% FCS/Dako buffer at four degrees overnight. Secondary antibodies, biotin conjugated donkey anti-rabbit or anti-mouse antibodies (Jackson Immuno Research), were applied 1:250 in 10% FCS/Dako buffer for 1h at room temperature (RT), detected by the Avidin-Biotin Complex Kit (ABC System, SP-2002, Vector Laboratories) and developed by diaminobenzidine staining (DAB plus Chromogen Solution, K3468, Dako). Cell nuclei were stained with Mayer’s Hemalum (Merck). Slides were scanned automatically (40x magnification) using a VS-120-L Olympus slide scanner 100-W system and pictures were processed using the Olympus VS-ASW-L100 software.

Quantification of HE and CD3^+^ cells was achieved by execution of automated image processing steps for whole area detection, immune cell detection and nuclei detection. Detection of whole section area in sections with CD3 stains was performed by an initial grayscale conversion of the image. Color information was converted to a representative 16-bit grey scale value by weighting the red, green and blue channels 1/3 each. A Sobel [6] edge detection algorithm was used to highlight inhomogeneous image areas, which represent the cell section, whereas homogeneous areas represent the image background. The application of a box blur [7] filter with a 9x9 kernel six times smoothed the sharp edges. Image regions with structural features represented higher intensity values, which could be detected by applying an intensity threshold of 50. Pixel artifact elimination was performed by filtering the objects with a minimum size of 1000 pixel and further blurring with a 19x19 filter kernel 26 times. A final threshold of 1 resulted in the discrimination of the entire histological section over background. Detection of the whole area on HE staining used similar pipelines with an initial TH of 10 and min particle size filter of 10000 pixel after Sobel edge detection. Additional blurring was done by applying an 11x11 filter kernel 10 times. Immune cell detection was achieved by initial brown tone color filtering followed by grey scale conversion of the filtered colors. For filtering, the HSV color [8] space was used with a hue range of 300 to 30, a saturation range of 30 to 100 and a brightness range of 70 to 170. A final threshold of 1 resulted in the discrimination of the immune cells over background. Nuclei detection was achieved by initial blue tone color filtering followed by grey scale conversion of the filtered colors. For filtering, the HSV color [8] space was used with a hue range of 195 to 259, a saturation range of 15 to 60 and a brightness range of 150 to 255. A final threshold of 1 resulted in the discrimination of the nuclei over background.

Heart slices for long-term survival. Antigen retrieval was performed by boiling sections in EDTA buffer for 20 minutes. Hematoxylin/Eosin staining was performed (Mayer’s Hemalum (MERCK) and Eosin Y (MERCK)) and collagen was visualized using Picrosirus Red staining (Abcam). Primary antibodies (Sup. Table 1) were applied in 1% PBS/BSA/AZIDE at room temperature (RT) for one hour or four degrees overnight in case of anti-N-cadherin antibody. As secondary antibody, one step detection system anti-Rabbit Alkaline Phosphate or anti-Mouse Alkaline Phosphate (BrightVision, ImmunoLogic) was applied at RT for 30 minutes followed with Liquid Permanent Red (Liquid Fast-Red Substrate System, Thermo Scientific™ Lab Vision™) and counterstaining with hematoxylin. For co-staining, sections were incubated with primary antibodies anti-Ku80 or anti-HLA Class I ABC at four degrees overnight and subsequently anti-Desmin at RT for one hour. Vector^®^ Blue Substrate Kit (Vector Laboratories) was used. Sections were mounted with medium and coverslips (ClearVue™ Mountant XYL, Thermo Scientific™, Thermo Fisher) and pictures were taken with a light microscope (Olympus). Infarct and graft areas were calculated from transverse sections, stained with SR and HLA Class I-Desmin co-stain using ImageJ software (1.47v).

**3D cryomicrotome imaging, pre-processing and segmentation**

*3D cryomicrotome imaging*

A whole heart from the long-term transplanted group was reserved for the 3D cryomicrotome imaging technique. This heart was embedded in a cylindrical holder in 3.0 % carboxymethylcellulose sodium solvent (Sigma) mixed with 0.1 % black ink (VWR), and frozen at -20°C for at least 24 hours. Three-dimensional optical imaging was performed using a custom-built automated imaging cryomicrotome (Department of Biomedical Engineering & Physics, Amsterdam UMC, The Netherlands).

In short, the sample was transversely cut in slices of 27.3 µm thickness after which the surface of the remaining bulk tissue was imaged with a 12-bit sCMOS camera (Kepler KL4040). Images of the fluorescent tracers were acquired using Luxeon LED modules in combination with an emission filter and labeled human microtissues were visualized using a 475 nm wavelength for excitation (Thorlabs MF475/35) and fluorescence was measured at 535 nm (Chroma, 535/50 nm) for emission; shutter time was set to 8000 ms. Reflection images were taken with an excitation and emission wavelengths of 510/20 nm; shutter time was set to 30 ms. All images were acquired using 1 x 1 binning, resulting in an in-plane resolution of 27.7 µm.

*3D image processing*

Image analysis pre-processing of the images was conducted to ensure the 3D image data fitted into the computer’s memory, and segmentation of relevant structures was conducted for visualization and volume quantification.

Pre-processing. The native cryomicrotome pictures contained 4096 x 4096 pixels, with a pixel spacing of 27.7 µm in both the X and Y direction. Gray-level values were stored as 16-bit values. The slice thickness was 27.34 µm and 3360 slices were made and imaged. These pictures were cropped to exclude the casting material and were subsequently scaled (20%) in the X and Y direction, and resampling in the Z-direction. This yielded a 3D image of (X, Y, Z) = (668 x 506 x 676) pixels with a uniform pixel spacing of 0.139 mm in all dimensions. Pre-processing was performed using custom software, written in the LabVIEW programming environment, fall 2019 version (National Instruments, Austin, Texas).

Segmentation. Image segmentation was performed as described by Dobbe et al.[9] In short, threshold-connected 3D region growing was repeatedly used to collect voxel regions above a given intensity threshold, starting at seed points chosen by the user. Remaining voxels were added manually using a digital bush to add voxels on a slice-by-slice basis, until the segmented region was visually approved by the observer. This intermediate result was used to initialize a Laplacian level-set segmentation growth algorithm,[10] which iteratively advanced the outline toward the location of the highest intensity gradient. Finally, the Marching cubes algorithm [11] was used to extract a polygon mesh at the zero-level of the level-set distance image.

**References**

[1] A. Haase, G. Göhring, and U. Martin, “Generation of non-transgenic iPS cells from human cord blood CD34+ cells under animal component-free conditions,” *Stem Cell Res*, vol. 21, pp. 71–73, May 2017, doi: 10.1016/J.SCR.2017.03.022.

[2] N. Kriedemann *et al.*, “Standardized production of hPSC-derived cardiomyocyte aggregates in stirred spinner flasks,” *Nature Protocols 2024*, pp. 1–29, Mar. 2024, doi: 10.1038/s41596-024-00976-2.

[3] G. P. van Hout *et al.*, “Admittance-based pressure-volume loops versus gold standard cardiac magnetic resonance imaging in a porcine model of myocardial infarction,” *Physiol Rep*, vol. 2, no. 4, p. e00287, 2014, doi: 10.14814/phy2.287.

[4] W. A. Gathier *et al.*, “Lower retention after retrograde coronary venous infusion compared with intracoronary infusion of mesenchymal stromal cells in the infarcted porcine myocardium,” *BMJ Open Sci*, vol. 3, no. 1, p. e000006, 2019, doi: 10.1136/bmjos-2018-000006.

[5] H. T. Van den Broek, L. De Jong, P. A. Doevendans, S. A. J. Chamuleau, F. J. Van Slochteren, and R. Van Es, “3D Whole-heart Myocardial Tissue Analysis,” *JoVE (Journal of Visualized Experiments)*, vol. 2017, no. 122, p. e54974, Apr. 2017, doi: 10.3791/54974.

[6] I. Sobel, “History and Definition of the so-called ‘Sobel Operator’, more appropriately named the Sobel-Feldman Operator.” Accessed: Dec. 31, 2024. [Online]. Available: https://www.scribd.com/document/271811982/History-and-Definition-of-Sobel-Operator

[7] J. Danmayr, “A reference implementation extracted from ImageJ using a 3x3 kernel.” Accessed: Dec. 31, 2024. [Online]. Available: https://github.com/joda01/imagec/blob/main/src/backend/commands/image_functions/blur/blur.cpp

[8] J. Danmayr, “A reference implementation for HSV colour filtering.” Accessed: Dec. 31, 2024. [Online]. Available: https://github.com/joda01/imagec/blob/main/src/backend/commands/image_functions/color_filter/color_filter.hpp

[9] J. G. G. Dobbe, M. G. A. de Roo, J. C. Visschers, S. D. Strackee, and G. J. Streekstra, “Evaluation of a Quantitative Method for Carpal Motion Analysis Using Clinical 3-D and 4-D CT Protocols,” *IEEE Trans Med Imaging*, vol. 38, no. 4, pp. 1048–1057, 2019, doi: 10.1109/TMI.2018.2877503.

[10] L. Ibanez, W. Schroeder, L. Ng, and J. Cates, “The ITK Software Guide: The Insight Segmentation and Registration Toolkit,” *IEEE Transactions on Information Technology in Biomedicine*, vol. 5, no. 4, p. 539, Sep. 2003, Accessed: Apr. 09, 2024. [Online]. Available: http://www.amazon.com/dp/1930934106

[11] W. E. Lorensen and H. E. Cline, “Marching cubes: A high resolution 3D surface construction algorithm,” *SIGGRAPH Comput. Graph.*, vol. 21, no. 4, pp. 163–169, 1987, doi: 10.1145/37402.37422.

**Supplementary videos**

**Supplementary video 1. Intramyocardial injection**

**Supplementary video 2. Matrigel^®^ plug implantation**

**Supplementary video 3. 3D heart reconstruction**

**Supplementary figures and tables**

**Supplementary Table 1. Antibody overview for flow cytometry and histology.**

| **Antibody** | **Host** | **Clonality** | **Clone #** | **Concentration** | **Company** |
| --- | --- | --- | --- | --- | --- |
| **Flow cytometry** | | | | | |
| Anti-pig CD3є | Mouse | Monoclonal | BB23-8E6-8c8 | 25 µg/mL | BD Pharmingen |
| Anti-pig CD4 | Mouse | Monoclonal | 74-12-4 | 2.5 µg/mL | BD Pharmingen |
| Anti-pig CD8a | Mouse | Monoclonal | 76-2-11 | 2.5 µg/mL | BD Pharmingen |
| **Histology** | | | | | |
| Anti-human HLA class I | Mouse | Monoclonal | EMR8-5 | 1 µg/mL | Abcam |
| Anti-human CD3 | Rabbit | Polyclonal |  | Ready-to-use | Dako |
| Anti-human CD56 | Mouse | Monoclonal | 123C3 | Ready-to-use | Ventana |
| Anti-human CD68 | Mouse | Monoclonal | KP1 | Ready-to-use | Dako |
| Anti-human CD138 | Mouse | Monoclonal | B-A38 | Ready-to-use | Cell marque |
| Anti-Ku80 | Rabbit | Monoclonal | EPR3468 | 0.6 ug/mL | Abcam |
| Anti-HLA Class 1 ABC | Mouse | Monoclonal | EMR8-5 | 2 ug/mL | Abcam |
| Anti-N-cadherin | Mouse | Monoclonal | C3865 | 2 ug/mL | Sigma |
| Anti-Porcine CD107a | Mouse | Monoclonal | 4E9/11 | 2 ug/mL | BioRad |
| Anti-Desmin | Mouse | Monoclonal | DE-R-11 | 0.1 mg/mL | Leica Biosystems |
| Anti-cardiac Troponin T | Rabbit | Polyclonal |  | 1 µg/mL | Abcam |


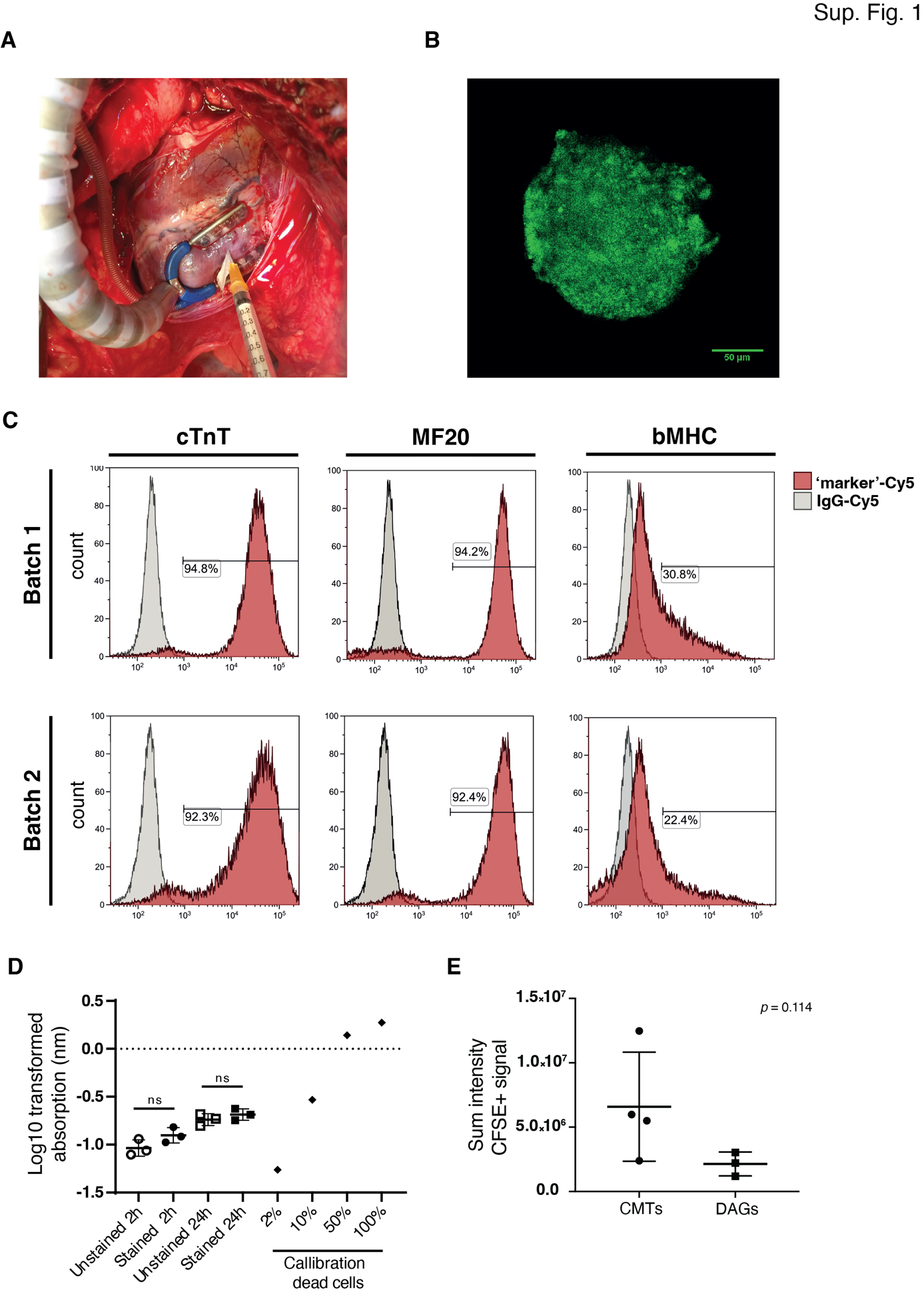
**Supplementary Figure 1. Characterization and acute retention evaluation of CMTs pre- and post-transplantation. (A)** Intramyocardial injections of CFSE-labeled CMTs and DAGs for acute retention evaluation. **(B)** Confocal image demonstrating CFSE staining in the core of the CMTs.  **(C)** Cardiomyocyte purity (%) assessment measured by flow cytometry for cTnT, MF20 and b-MHC after hiPSC-CM differentiation in bioreactors. Pink peaks, antibody of interest; gray peaks, IgG isotype control. **(D)** Cell viability (% dead) of CMTs 2h or 24h after CFSE staining and unstained CMTs under same culture conditions measured with a LDH cytotoxicity assay. Cell death was calculated from Log10 transformed absorption values from known cell death percentages. **(E)** Quantification of sum CFSE positive signal intensity of heart slices from hearts transplanted with CMTs and DAGs. Panel (D): one-way ANOVA test (*n* = 3 biological replicates); data are mean ± SD. Panel (E): Mann-Whitney test (*n* = 3-4 biological replicates); data are mean ± SD. cTnT, cardiac troponin T; CFSE, carboxyfluorescein succinimidyl ester; CMTs, cardiac microtissues; MF20, myosin heavy chain; b-MHC, beta myosin heavy chain.

**Supplementary Table 2. Circulating levels of immunosuppressants during optimization and Matrigel^®^ plugs transplantation.**

|  | | ***Dose given*** | | ***Concentration measured*** | | | | | |
| --- | --- | --- | --- | --- | --- | --- | --- | --- | --- |
|  |  | ***Tacrolimus***  *(mg/kg/day)* | ***Azathioprine***  *(mg/kg/day)* | ***Tacrolimus***  *µg/L* | | | ***Azathioprine (6-TGN)*** *(pmol)/8x10^8^ RBC* | | |
|  |  |  |  | ***#1*** | ***#2*** | ***#3*** | ***#1*** | ***#2*** | ***#3*** |
| **Optimization (week)** | **1** | 0.0 | 0.0 | <2 | <2 | <2 | <50 | <50 | <50 |
|  | **2** | 0.35 | 1.0 | 3.2 | 4.9 | 3.2 | <50 | <50 | <50 |
|  | **3** | 0.35 | 2.0 | 2.7 | x | 2.5 | <50 | x | <50 |
|  | **4** | 0.4 | 2.0 | 2.5 | 4.5 | 2.5 | <50 | <50 | <50 |
|  | **5** | 0.7 | 4.0 | 5.4 | 3.2 | 2.3 | <50 | <50 | 62 |
|  | **6** | 0.7 | 4.0 | 4.1 | 6.5 | 4.0 | <50 | <50 | <50 |
|  | **7** | 0.7 | 4.0 | 6.4 | 6.8 | 3.9 | 52 | <50 | 60 |
|  | **8** | 0.7 | 4.0 | 6.6 | 7.3 | 4.5 | <50 | <50 | 80 |
|  | **9** | 0.7 | 4.0 | x | x | 6.9 | x | x | 76 |
|  | **10** | 1.0 | 5.0 | 10.0 | 12.3 | 9.2 | 64 | <50 | 109 |
|  | **11** | 1.0 | 5.0 | 12.0 | 13.3 | 11.4 | 60 | <50 | 122 |
|  | **12** | 1.0 | 7.0 | 11.7 | 17.9 | 12.2 | 64 | 51 | 151 |
|  | **13** | 1.0 | 7.0 | 16.2 | 14.7 | 10.4 | 64 | 55 | 180 |
| **Transplantation (day)** | **0** | 1.0 | 7.0 | 10.7 | 23.6 | 9.9 | 74 | 92 | 206 |
|  | **3** | 1.0 | 7.0 | 14.5 | 24.9 | 13.5 | 63 | 97 | 213 |
|  | **7** | 1.0 | 7.0 | 13.3 | x | 8.8 | <50 | x | 231 |
|  | **9** | 1.0 | 7.0 | 11.4 | 18.9 | 9.0 | <50 | x | 245 |
|  | **14** | 1.0 | 7.0 | 5.7 | 12.8 | 13.2 | <50 | 95 | 213 |

**Supplementary Table 3.** Blood biochemistry parameters measured in healthy pigs

|  | ***Concentration measured*** | | | | | | | |
| --- | --- | --- | --- | --- | --- | --- | --- | --- |
|  | ***ASAT***  *U/L* | | ***ALAT***  *U/L* | | ***γ-GT***  *U/L* | | ***Creatinine***  *µmol/L* | |
|  | **No IS**  **(*n* = 2)** | **IS**  **(*n* = 3)** | **No IS**  **(*n* = 2)** | **IS**  **(*n* = 3)** | **No IS**  **(*n* = 2)** | **IS**  **(*n* = 3)** | **No IS**  **(*n* = 2)** | **IS**  **(*n* = 3)** |
| **Baseline** | 44.5 ± 12.02 | 61.33 ± 9.452 | 77.5 ± 24.75 | 110.7 ± 29.09 | 41 ± 2.828 | 68.67 ± 44.5 | 146.5 ± 3.536 | 108 ± 23.9 |
| **Termination** | 43 ± 12.73 | 42 ± 11.36 | 77.5 ± 33.23 | 61 ± 10.39 | 40 ± 9.899 | 32.67 ± 13.65 | 131 ± 12.73 | 132.7 ± 20.03 |


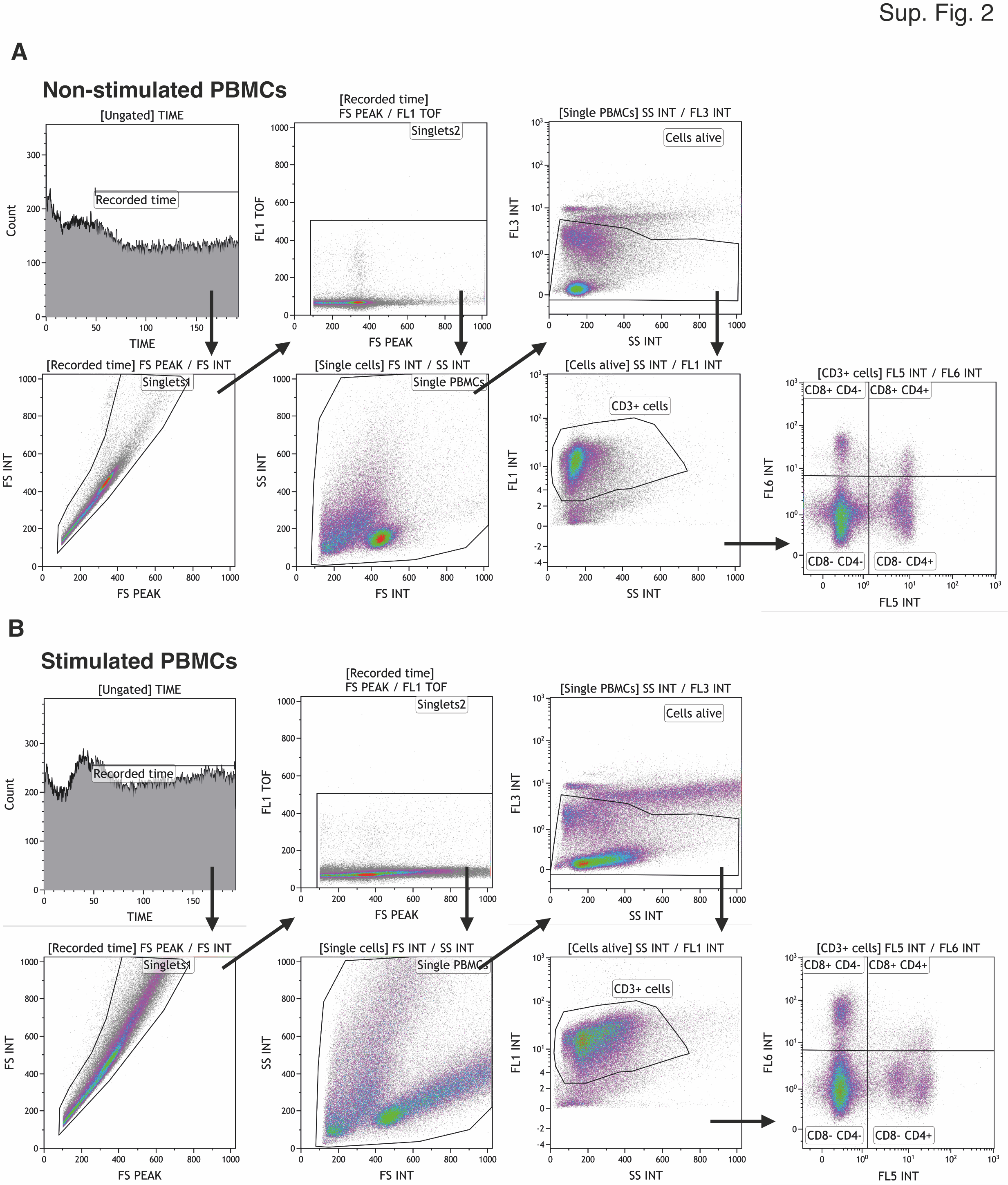
**Supplementary Figure 2.** **Gating strategy of T cell subsets.** Gating strategy for **(A)** non-stimulated and **(B)** stimulated PBMCs. After doublet exclusion, PBMCs were distinguished based on size and granularity and viable cells were selected. Based on surface expression of CD3, we selected CD3^+^, CD4^+^ and CD8^+^ T cell subsets. Panel (A): (*n* = ~3×10^5^ biological replicates). Panel (B): (*n* = ~3×10^5^ biological replicates). PBMCs, peripheral blood mononuclear cells.


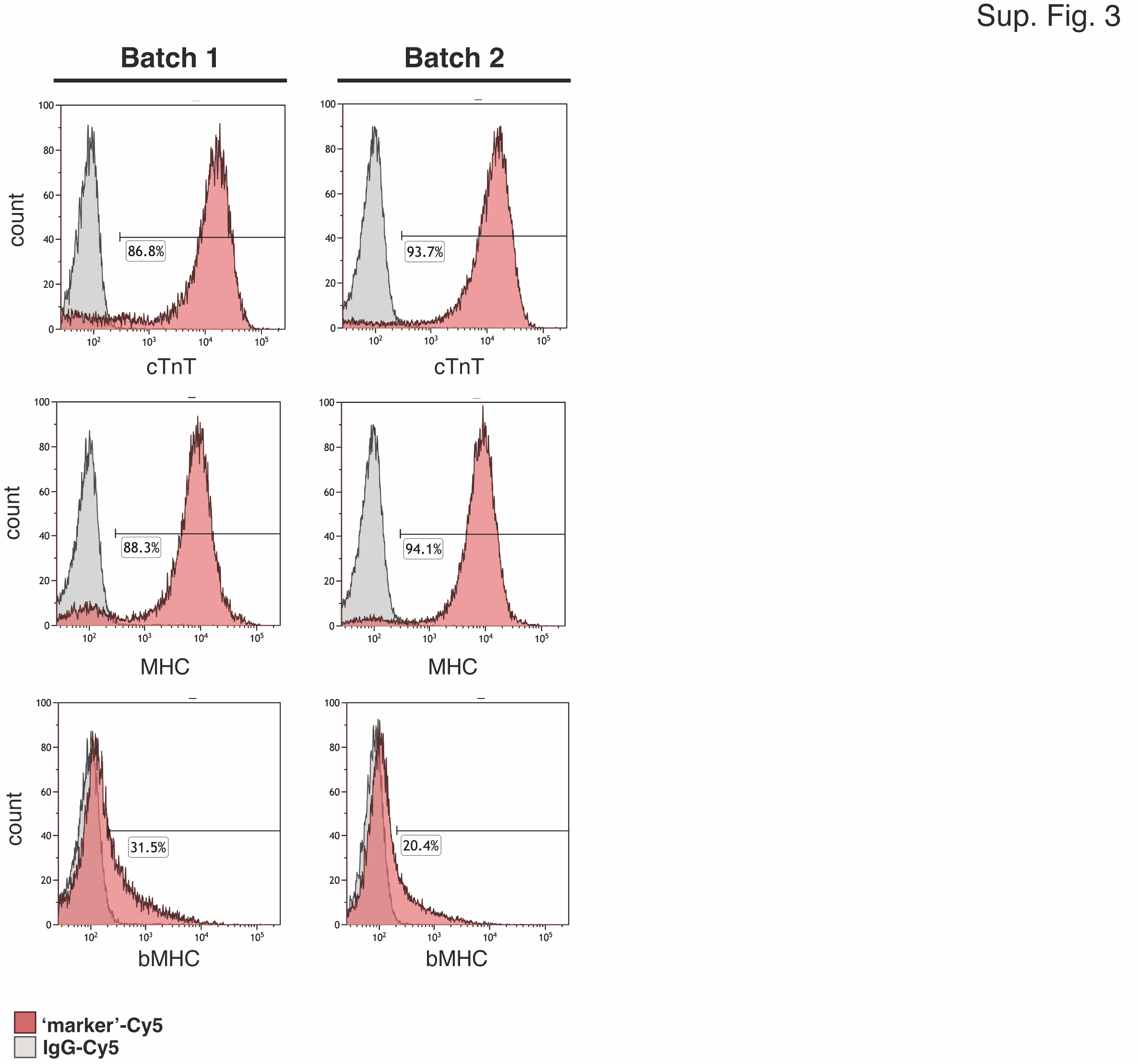


**Supplementary Figure 3. Cardiomyocyte purity (%) assessment for cardiac xenotransplantation during immunosuppression optimization.** Markers for cTnT, MHC and the maturation b-MHC after hiPSC-CM differentiation in bioreactors measured by flow cytometry. cTnT and MHC assessment reveals the efficient, highly reproducible induction of ~90% CMs in individual differentiation batches (*n* = ~1.5×10^5^ biological replicates). Pink peaks, antibody of interest; gray peaks, IgG isotype control. cTnT, cardiac troponin T; MF20, myosin heavy chain; b-MHC, beta myosin heavy chain.


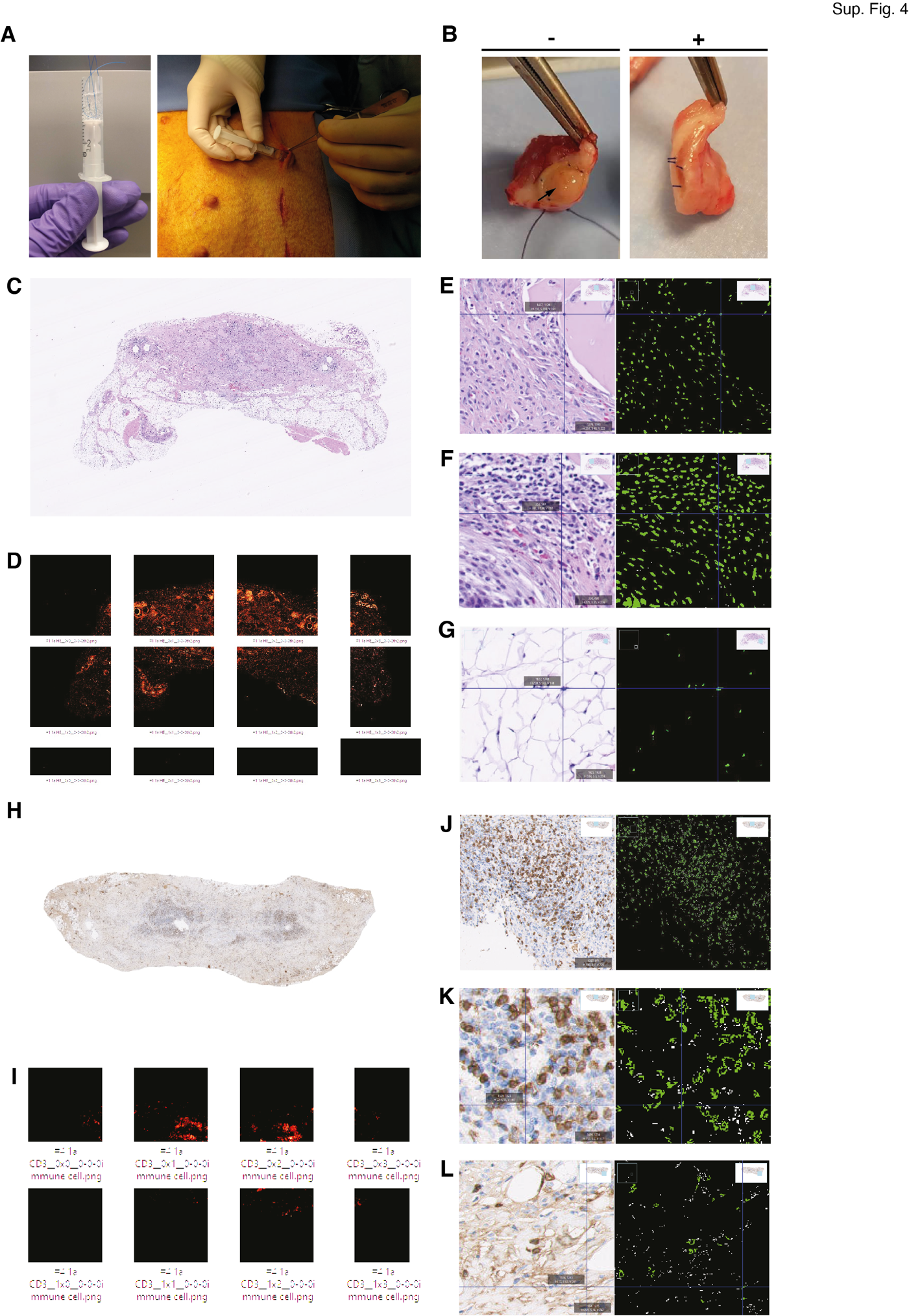
**Supplementary Figure 4. Host cell infiltrate upon CMT transplantation in Matrigel^®^ plugs. (A)** Human donor cell-loaded Matrigel^®^ plug preparation and subcutaneous transplantation. **(B)** Macroscopic images of the plugs during recovery showed ulcerous tissue (black arrow) in No-IS pigs, absent in IS pigs. **(C-L)** Using an automated imaging processing strategy, general HE staining **(C-G)** and CD3^+^ cell infiltrates **(H-L)** were quantified. Exemplary images of the original micrography are shown in **(C)** and **(H)**, with an overview of immune cell detection **(D and I)**. Detail images with different magnifications are shown for different cell areas and immune cells in HE **(E-G)** and CD3^+^ **(J-L)** stainings. Nuclear counts from HE images (purple nuclei) and CD3^+^ positive cells (dark brown) are displayed as green areas and spots that are not considered for quantification are indicated in white. IS (+), immunosuppressed; No-IS (-), non-immunosuppressed.


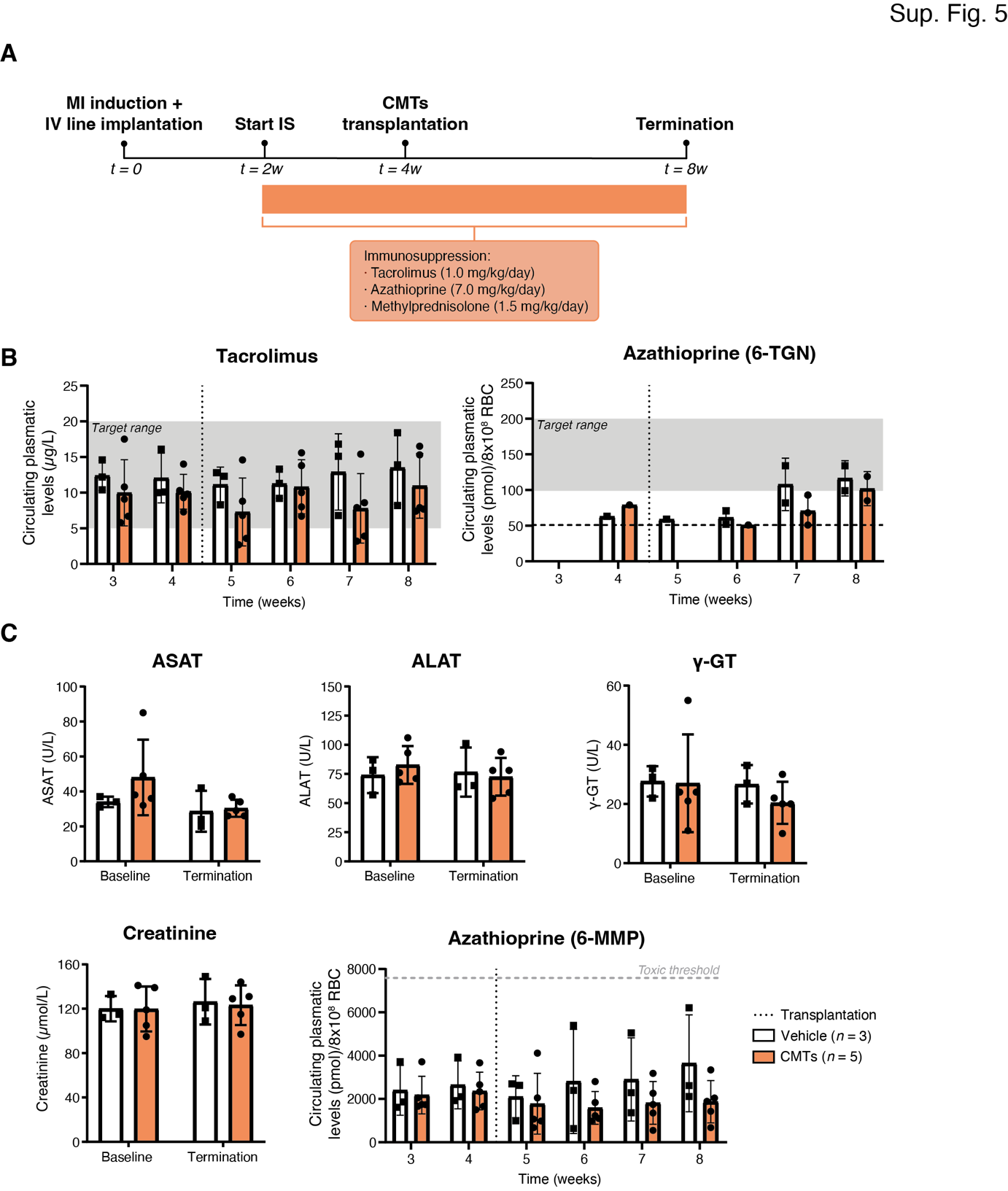
**Supplementary Figure 5. Study design, drug concentrations, and biochemistry in pigs. (A)** Study design and timeline of the experiment. **(B)** Circulating tacrolimus and azathioprine 6-TGN concentrations prior and post xenotransplantation. Lower detection limit 6-TGN (> 50 pmol 6-TGN/8x10^8^ RBC) is represented by horizontal dashed line. **(C)** Serum concentrations of routine clinical chemistry parameters for kidney and liver function at MI induction (baseline) and termination. Azathioprine 6-MMP levels were also measured prior and post-xenogeneic CMT transplantation. Panels (B and C): vehicle (*n* = 3 biological replicates); CMTs (*n* = 5 biological replicates). Data are mean ± SD. ALAT, alanine aminotransferase; ASAT, aspartate aminotransferase; CMTs, cardiac microtissues; IS, immunosuppression; *i.v.*, intravenous; MI, myocardial infarction; RBC, red blood cells; t, timepoint; w, week; ɣ-GT, gamma-glutamyl transferase; 6-MMP, 6-methylmercaptopurine nucleotide; 6-TGN, 6-thioguanine nucleotide.

**Supplementary Table 4. Dosing and circulating levels of immunosuppressants in infarcted pigs.**

|  | | ***Concentration measured*** | | | | | | | | | | | | | | | |
| --- | --- | --- | --- | --- | --- | --- | --- | --- | --- | --- | --- | --- | --- | --- | --- | --- | --- |
|  |  | ***Tacrolimus^a^***  *µg/L* | | | | | | | | ***Azathioprine (6-TGN)^a^***  *(pmol)/8x10^8^ RBC* | | | | | | | |
|  |  | ***#1*** | ***#2*** | ***#3*** | ***#4*** | ***#5*** | ***#6*** | ***#7*** | ***#8*** | ***#1*** | ***#2*** | ***#3*** | ***#4*** | ***#5*** | ***#6*** | ***#7*** | ***#8*** |
| **Relative to transplantation (day)** | **-14** | <1.3 | <1.3 | <1.3 | <1.3 | <1.3 | <1.3 | <1.3 | <1.3 | <50 | <50 | <50 | <50 | <50 | <50 | <50 | <50 |
|  | **-7** | 17.5 | 12.2 | 9.1 | 14.6 | 10.4 | 10.8 | 6.7 | 5.8 | <50 | <50 | <50 | <50 | <50 | <50 | <50 | <50 |
|  | **0** | 14 | 10 | 9.3 | 16.1 | 10.1 | 9.8 | 10.3 | 7.3 | <50 | 63 | 79 | <50 | <50 | <50 | <50 | <50 |
|  | **7** | 14.6 | 8.3 | 8.8 | 12.3 | 12.8 | 3.6 | 6.8 | 2.7 | <50 | 59 | <50 | <50 | <50 | <50 | <50 | <50 |
|  | **14** | 13.8 | 9.2 | 15.6 | 11.3 | 13.3 | 6.7 | 10 | 8 | <50 | 71 | <50 | <50 | 52 | <50 | <50 | 51 |
|  | **21** | 15.4 | 6.8 | 7.9 | 16.8 | 15.1 | 3.2 | 3.9 | 8.6 | 51 | 134 | 68 | <50 | 82 | <50 | <50 | 93 |
|  | **28** | 16.5 | 8.2 | 7.9 | 18.4 | 13.9 | 7.4 | 15.3 | 7.7 | <50 | 134 | 119 | <50 | 99 | <50 | <50 | 85 |

^a^ Dose given: 1.0 mg/kg/day of tacrolimus and 7.0 mg/kg/day of azathioprine.

**Supplementary Table 5.** Blood biochemistry parameters measured in infarcted pigs

|  | ***Concentration measured*** | | | | | | | |
| --- | --- | --- | --- | --- | --- | --- | --- | --- |
|  | ***ASAT***  *U/L* | | ***ALAT***  *U/L* | | ***γ-GT***  *U/L* | | ***Creatinine***  *µmol/L* | |
|  | **Saline**  **(*n* = 3)** | **CMT**  **(*n* = 5)** | **Saline**  **(*n* = 3)** | **CMT**  **(*n* = 5)** | **Saline**  **(*n* = 3)** | **CMT**  **(*n* = 5)** | **Saline**  **(*n* = 3)** | **CMT**  **(*n* = 5)** |
| **Baseline** | 34  ± 3 | 48  ± 21.62 | 74  ± 15.39 | 82.8  ± 16.18 | 27.67 ± 5.132 | 27  ± 16.54 | 120  ± 11.36 | 119.8 ± 20.33 |
| **Termination** | 28.67 ± 11.72 | 30.40  ± 4.93 | 76.67 ± 21.08 | 72.6  ± 16.18 | 26.67 ± 6.506 | 20.4  ± 7.127 | 126.3 ± 20.53 | 123.2 ± 17.89 |


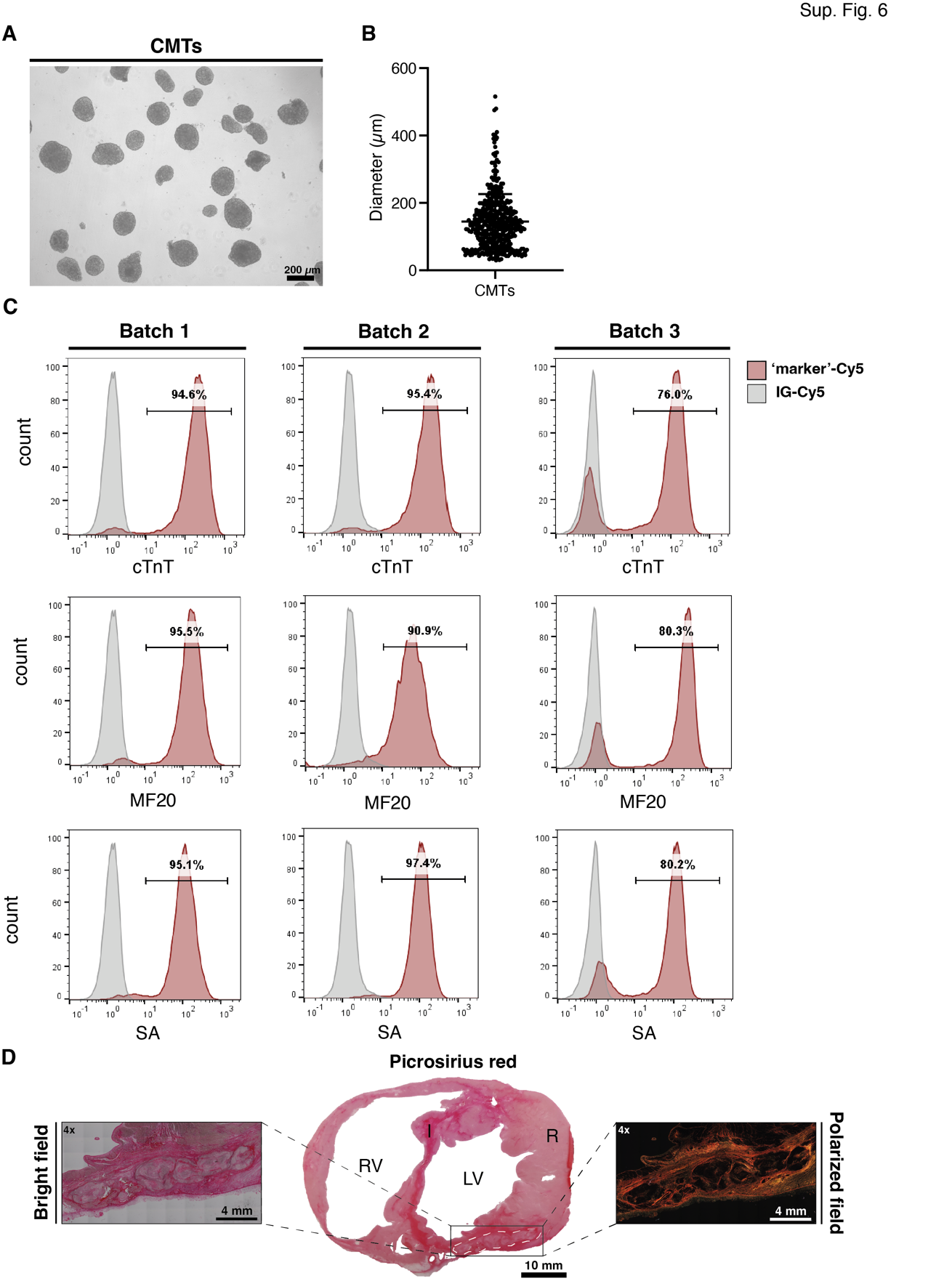
**Supplementary Figure 6. CMT assessment by means of flow cytometry and histology. (A)** Representative bright field images of CMTs. **(B)** Quantification of CMTs diameter pre-transplantation. **(C)** Markers for cTNT, MF20 and SA after hiPSC-CM differentiation in bioreactors measured by flow cytometry. Pink peaks, antibody of interest; gray peaks, IgG isotype control. **(D)** Bright field and birefringent images of a representative picrosirius red staining were used to visualize the collagen scar in the heart cross-section transplanted with CMTs. Scale bar heart section = 10 mm; scale bar 4x = 4 mm. Panel (B): *n* = 437 biological replicates; data are mean ± SD. Panel (C): *n* = 1.5×10^5^ biological replicates. Panel (D): representative image of a CMT-transplanted infarcted heart (*n* = 1 biological replicate). CMTs, cardiac microtissues; DAGs, dissociated aggregates; CFSE: carboxyfluorescein succinimidyl ester. CMTs, cardiac microtissues; cTnT, cardiac troponin T; I, infarcted myocardium; LV, left ventricle; MF20, myosin heavy chain; R, remote myocardium; RV, right ventricle; SA, sarcomeric a-actinin; white dashed line, transplanted CMTs.

**
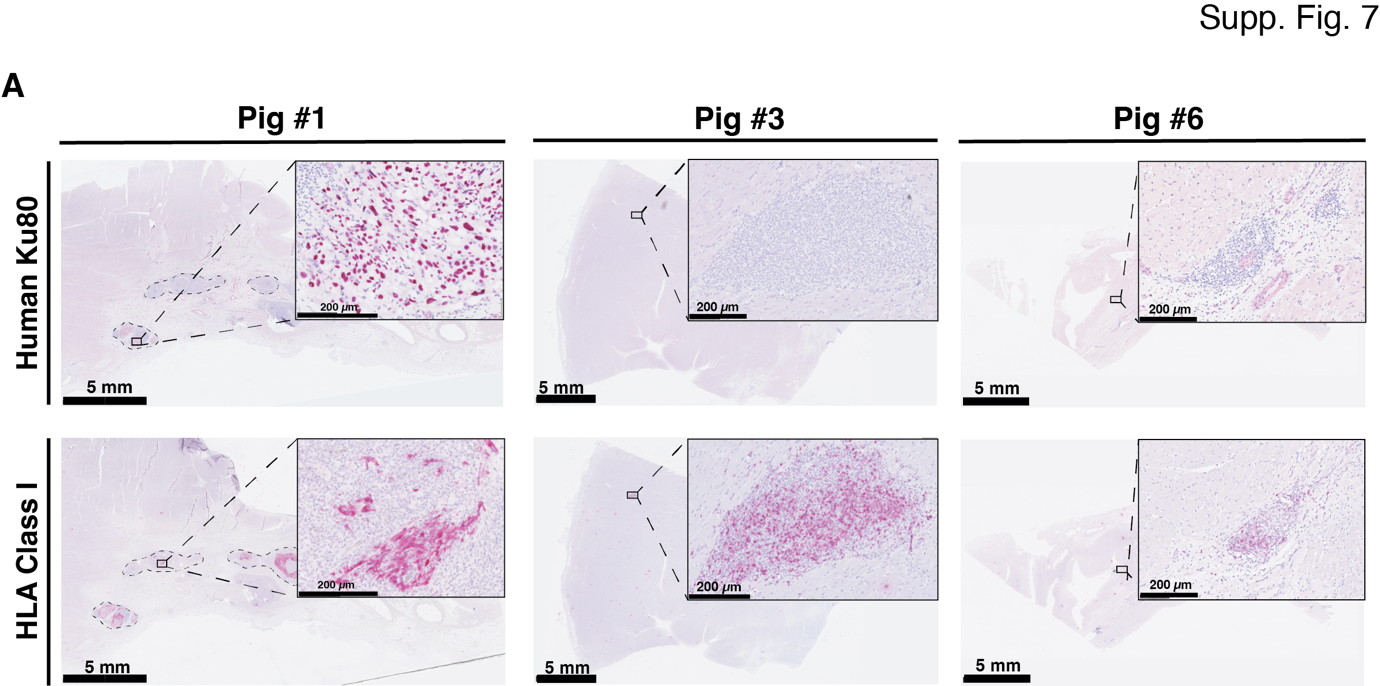
**

**Supplementary Figure 7. Human cell retention four weeks post-CMT transplantation.** Heart sections from four transplanted pigs were created to detect the presence of CMTs using the human-specific nuclear marker Ku80 (pink) and HLA class I (pink). Positive staining for human markers were found in three out of four animals, although only large grafts were seen in one and taken as a positive control (pig #1). Scale bar micrograph section = 5 mm; scale bar zoomed in micrograph = 200 µm. Micrographs are representative images from CMT-transplanted hearts (*n* = 3 biological replicates). Black square, zoomed in area; dashed lines, CMTs.


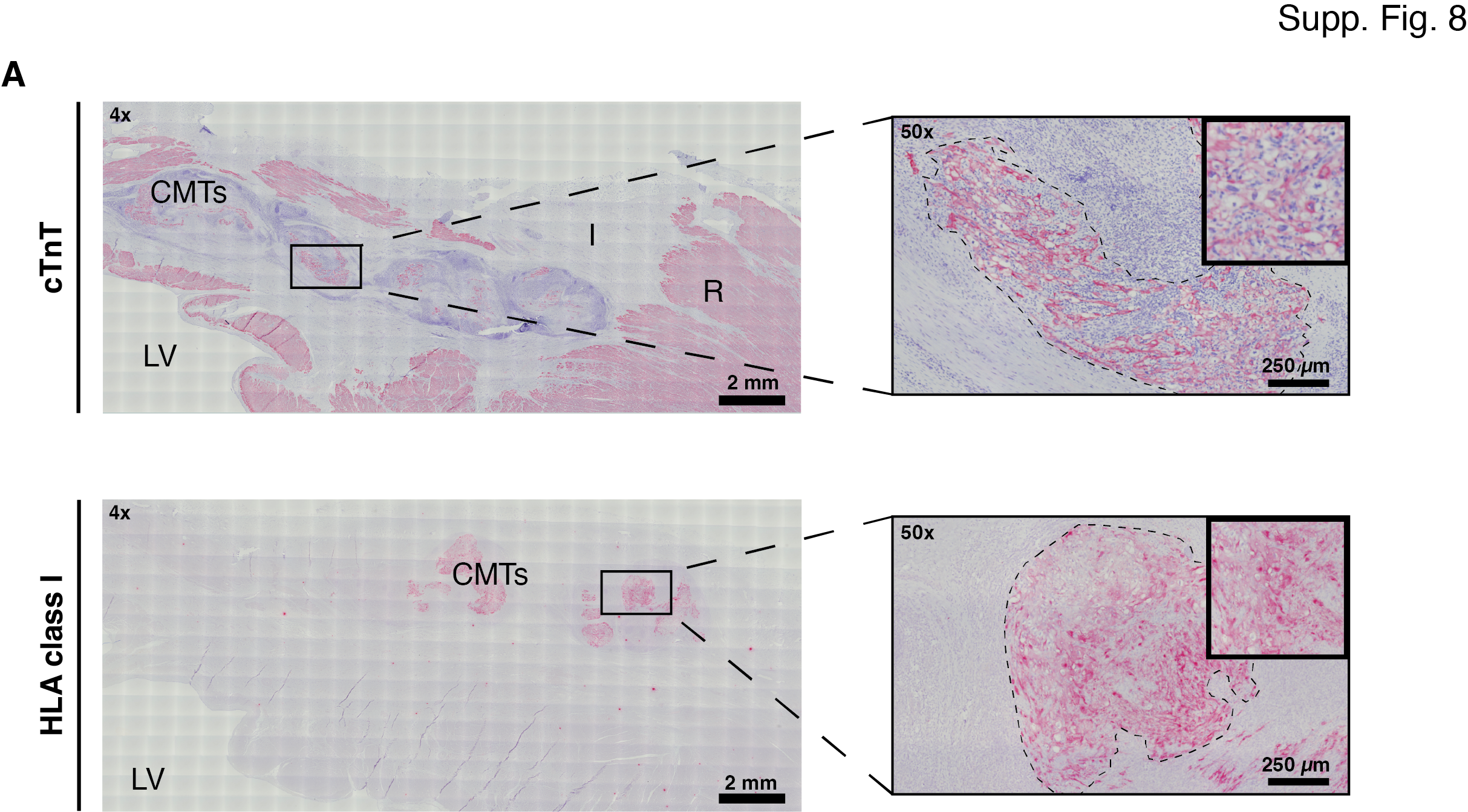


**Supplementary Figure 8. Infarct remuscularization four weeks post-CMT transplantation.** Heart sections of a heart four weeks after CMT-transplantation stained with cardiac troponin T (top image, pink) and HLA class I marker (bottom image, pink) proving that human grafts (black dashed lined) remain differentiated into CM. Scale bar 4x = 2 mm; scale bar 50x = 250 µm. Micrographs are representative images from a CMT-transplanted heart (*n* = 1 biological replicate). CMTs, cardiac microtissues; I, infarcted myocardium; LV, left ventricle; R, remote myocardium.

**
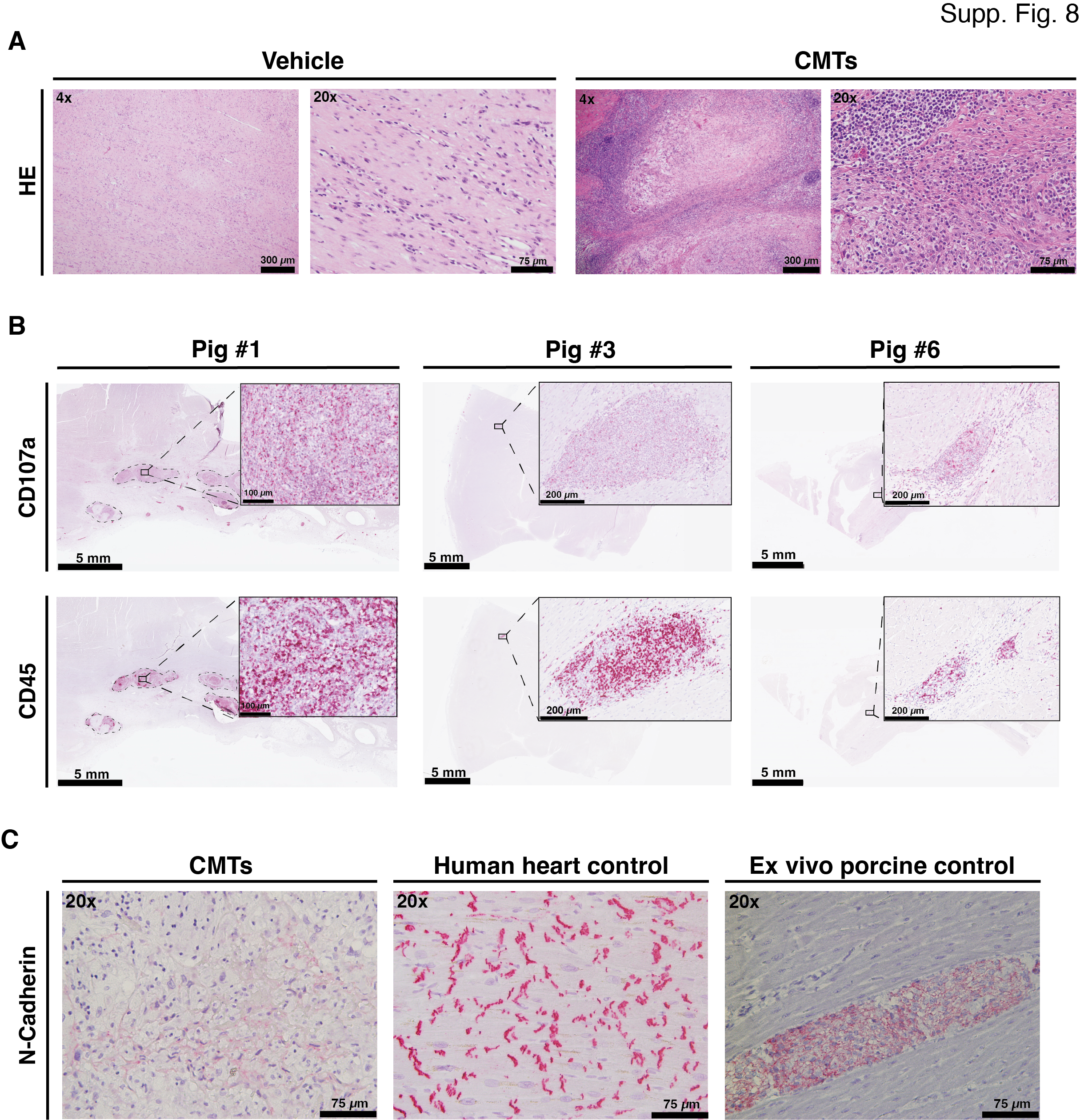
Supplementary Figure 9. Host cell infiltration and integration marker in CMT-transplanted area. (A)** Visualization of representative HE staining of infarct scar in control (vehicle) and CMT-transplanted pigs. **(B)** Immune infiltration assessment with porcine-specific CD107a (pink) and general immune infiltration CD45 (pink) markers around the CMT area. **(C)** Loss of intercalated disk marker N-cadherin (pink) in one CMT-transplanted pig four weeks after transplantation. N-cadherin was present in a control human heart and after *ex vivo* CMT transplantation in porcine cardiac tissue control. Panels (A and C): scale bar 4x = 300 µm; scale bar 20x = 75 µm. Panel (B) Scale bar heart section = 5 mm; scale bar micrograph = 200 µm. CMTs, cardiac microtissues; HE, hematoxylin/eosin.
